# Immune cell senescence and exhaustion promote the occurrence of liver metastasis in colorectal cancer by regulating epithelial-mesenchymal transition

**DOI:** 10.18632/aging.205778

**Published:** 2024-04-26

**Authors:** Sen Lin, Lanyue Ma, Jiaxin Mo, Ruiqi Zhao, Jinghao Li, Mengjiao Yu, Mei Jiang, Lisheng Peng

**Affiliations:** 1 The Fourth Clinical Medical College, Guangzhou University of Chinese Medicine, Guangzhou, China; 2 The First Clinical Medical College, Guangzhou University of Chinese Medicine, Guangzhou, China; 3 Department of Traditional Chinese Medicine, The Sixth Affiliated Hospital, South China University of Technology, Foshan, China; 4 Department of Oncology, The First Affiliated Hospital, Guangzhou University of Chinese Medicine, Guangzhou, China; 5 Department of Hepatology, Shenzhen Traditional Chinese Medicine Hospital, Shenzhen, China

**Keywords:** senescence, exhaustion, colorectal cancer liver metastases, epithelium-mesenchymal transition, machine learning

## Abstract

Background: Liver metastasis (LM) stands as a primary cause of mortality in metastatic colorectal cancer (mCRC), posing a significant impediment to long-term survival benefits from targeted therapy and immunotherapy. However, there is currently a lack of comprehensive investigation into how senescent and exhausted immune cells contribute to LM.

Methods: We gathered single-cell sequencing data from primary colorectal cancer (pCRC) and their corresponding matched LM tissues from 16 mCRC patients. In this study, we identified senescent and exhausted immune cells, performed enrichment analysis, cell communication, cell trajectory, and cell-based *in vitro* experiments to validate the results of single-cell multi-omics. This process allowed us to construct a regulatory network explaining the occurrence of LM. Finally, we utilized weighted gene co-expression network analysis (WGCNA) and 12 machine learning algorithms to create prognostic risk model.

Results: We identified senescent-like myeloid cells (SMCs) and exhausted T cells (TEXs) as the primary senescent and exhausted immune cells. Our findings indicate that SMCs and TEXs can potentially activate transcription factors downstream via ANGPTL4-SDC1/SDC4, this activation plays a role in regulating the epithelial-mesenchymal transition (EMT) program and facilitates the development of LM, the results of cell-based *in vitro* experiments have provided confirmation of this conclusion. We also developed and validated a prognostic risk model composed of 12 machine learning algorithms.

Conclusion: This study elucidates the potential molecular mechanisms underlying the occurrence of LM from various angles through single-cell multi-omics analysis in CRC. It also constructs a network illustrating the role of senescent or exhausted immune cells in regulating EMT.

## INTRODUCTION

Colorectal cancer (CRC) is a malignant tumor disease that poses a significant threat to human health, with high rates of mortality and disability. Recent epidemiological report [1] indicates that CRC is the second leading cause of death from malignant tumors, surpassed only by lung cancer. Despite the fact that early screening for CRC in high-risk populations has not been fully implemented, approximately 20–25% of new cases are diagnosed as metastatic colorectal cancer (mCRC). Furthermore, about 30% of early and middle-stage cases experience postoperative recurrence after radical surgery. The 5-year survival rate for mCRC patients is less than 5% [2, 3]. Liver metastasis (LM) is the primary cause of death in mCRC patients, with about 80–90% of mCRC patients developing LM. Currently, the treatment approach for mCRC combines targeted therapy with immunotherapy. However, as CRC is considered a ‘cold tumor’ with low immunogenicity [4], less than 5% of patients benefit from immunotherapy, which does not significantly improve the poor survival rate of mCRC [5].

The progression, metastasis, and resistance to immune therapy of malignant tumors are closely associated with immune cell dysfunction, primarily characterized by senescence and exhaustion. Both senescent and exhausted immune cells exhibit characteristics of late cell development but promote the progression of malignant tumors through different mechanisms [6, 7]. Senescent cells secrete typical senescence associated secretory phenotype (SASP) [8], including factors such as IL-6, IL-10, AREG. These factors promote the progression and deterioration of malignant tumors through various pathways such as promoting epithelial-mesenchymal transition (EMT) [9, 10], inducing tumor cell stemness [11], immune suppression [12, 13], inducing carcinogenesis [14], promoting metastasis [15], and drug resistance [16]. Exhausted immune cells primarily affect the progression and treatment effect of malignant tumors by regulating the tumor microenvironment (TME) [17]. They express multiple inhibitory receptors such as PD-1, CTLA-4, TIM-3, and TIGIT and form a complex inhibitory TME through crosstalk and interaction with stromal cells, tumor cells, and various secretory factors. This leads to immune cells losing their ability to recognize, monitor, and kill tumor cells [18, 19].

Currently, the potential mechanism by which senescent and exhausted immune cells promote LM in CRC has not been fully elucidated. Although senescence [20] and exhaustion [21] represent two distinct types of immune cell dysfunction, they both promote the progression of malignant tumors through multiple mechanisms. With the advent of single-cell sequencing technology, it has become a new direction for malignant tumor research. Single-cell multi-omics analysis integrates metabolic analysis, cell communication, cell differentiation trajectory analysis from genomics, transcriptomics, metabolomics, and TME aspects to perform multi-dimensional analysis on the status and fate of individual cells. Therefore, this study explores the potential mechanism by which senescent and exhausted immune cells promote LM in CRC through single-cell multi-omics analysis. In combination with machine learning algorithms and clinical data to construct a prognostic risk model provides a new direction for the development of clinical drugs for mCRC.

## MATERIALS AND METHODS

### Data acquisition and preprocessing

The single-cell RNA sequencing (scRNA-Seq) data employed in this study originate from two publicly available datasets of single-cell sequencing research [22, 23]. The sequencing platform utilized was the Illumina NovaSeq 6000. The scRNA-Seq data from primary colorectal cancer (pCRC) and their matched liver metastasis (LM) tissues underwent separate preprocessing using the Seurat package (v4.3.0) [24] in R (v4.2.2). This entailed data quality control and SC-Transform normalization. Subsequently, the harmony package (v0.1.1) in R [25] was applied to integrate samples and correct batch effects.

### Dimensionality reduction and clustering

Principal Component Analysis (PCA) was conducted on the preprocessed scRNA-Seq data, and the Louvain algorithm was employed for modularity optimization. The clustree package (v0.5.0) in R [26] was used to determine an appropriate resolution (0.5–1.2) for defining cell clusters. Following this, UMAP [27] was utilized to further reduce the dimensionality of PCA-reduced data, and the results of dimensionality reduction clustering were visualized.

### Cell annotation and cell trajectory analysis

Differential gene expression analysis for each cell cluster was conducted using the Wilcoxon rank-sum test, and differentially expressed genes (DEGs) for each cell type were identified based on Log2Fc >0.5 and Benjamini–Hochberg-adjusted *p*-values < 0.05. The cell clusters obtained from dimensionality reduction clustering were annotated based on these DEGs. Automatic annotation and manual corrections were carried out using the SingleR package (v1.10.0) in R [28], classifying the cell clusters into six main cell types.

To identify senescence cell subtypes, cell trajectory analysis was performed. The Monocle3 package (v1.3.1) [29–31] in R was employed for pseudotime analysis, utilizing the SimplePPT algorithm for trajectory learning. An iterative algorithm for semi-supervised pseudotime analysis was used to construct a developmental trajectory map of cells. Spatial differential gene algorithms calculated Moran’s I of genes, with Moran’s I values ranging between -1 and 1, where 1 indicates a high positive correlation, and Moran’s I less than or equal to 0 indicates no correlation. This analysis identified development-related genes based on Moran’s I.

For each main cell type, the steps of dimensionality reduction clustering and differential gene expression analysis were repeated, and manual annotation was performed based on the expression characteristics of DEGs combined with the results of cell trajectory analysis to identify senescent and exhausted immune cells, annotating epithelial cells, myeloid cells, and NK/T cells into different cell subtypes.

### Enrichment analysis

To explore differences in function, signaling pathways, and metabolic activity between senescent and exhausted immune cells in pCRC and LM tissues, senescent-like myeloid cells (SMCs) were extracted and merged from myeloid cells in pCRC and LM tissues for subsequent enrichment analysis. Differential gene expression analysis was performed on cell subtypes from different tissue sources to obtain DEGs for enrichment analysis.

First, gene set enrichment analysis (GSEA) was conducted on cell subtypes from different tissue sources using the fgsea package (v1.22.0) [32] in R, with Hallmark serving as the gene set for signaling pathways. Subsequently, enrichment analysis was performed based on biological function gene set Gene Ontology (GO) and signaling pathway gene set Kyoto Encyclopedia of Genes and Genomes (KEGG). The results were presented as a bubble plot. Metabolic activity analysis was carried out using the scMetabolism package (v2.1.0) [33] in R, primarily to assess differences in metabolic pathway activity between cell subtypes in pCRC and LM tissues, with KEGG and Reactome as the gene sets for metabolic pathways.

### Cell communication analysis

Myeloid cells from pCRC and LM tissues, as well as NK/T cells, were extracted and merged for cell communication analysis. The CellChat package (v1.6.1) [34] in R was utilized to infer ligand-receptor pair communication between different cell subtypes and create a cell communication network diagram. Through CellChat algorithm calculations, outgoing or incoming signal strength was compared to assess the overall signal flow strength of different secretory factors in pCRC and LM tissues. The focus was on crosstalk between tumor cells and SMCs or TEXs to identify potential ligand-receptor pairs.

### *In vitro* validation experiments

Building upon our preceding analysis of cell communication, we identified notably active ANGPTL4-SDC1/SDC4 ligand-receptor pairs within the SMCs. To establish the connection between the expression of surface receptors, SDC1 and SDC4, and the process of EMT, a series of *in vitro* experiments was undertaken.

Initially, we conducted wound healing tests on HCT116 cells, employing SDC1 or SDC4 knockdown. The extent of wound healing was assessed at 0 h, 24 h, and 48 h, and wound healing percentages were calculated using ImageJ Software (v1.54d). Subsequently, Transwell migration assays were carried out to evaluate the migratory capacity of HCT116 cells after the knockdown of SDC1 or SDC4.

RT-qPCR was conducted to measure the expression levels of EMT-related markers E-Cadherin (CDH1), N-Cadherin (CDH2), and Vimentin (VIM) in HCT116 cells following siRNA knockdown of SDC1 and SDC4. A negative control group (si-NC) and HCT116 cells transfected with si-SDC1 or si-SDC4 were cultured for 48 hours, and RNA was extracted. After cell lysis with Trizol, RNA was separated and extracted with a washing solution. cDNA was synthesized, and RT-qPCR was performed to detect EMT-related marker expression levels. The relative mRNA expression level of EMT-related markers was calculated by the 2^−ΔΔCt^ method, and the expression level difference between si-NC and si-SDC1/si-SDC4 was compared by unpaired *t*-test (*P* < 0.05 was considered statistically significant). The sequences of si-SDC1 and si-SDC4, as well as the primer sequences of EMT-related markers (CDH1, CDH2, and VIM) and reference genes (β-Actin), can be found in Supplementary Tables 1–4.

According to the RT-qPCR experiment grouping mentioned above, the expression levels of EMT-related proteins E-Cadherin, N-Cadherin, and Vimentin in HCT-116 cells were detected by Western blot. Total protein was extracted from the samples using cell lysis buffer, and the protein mass and concentration were determined using a protein quantification kit (BCA method). Gels were prepared using 10% or 15% PAGE gels, and an appropriate amount of electrophoresis buffer was added to the electrophoresis tank. A suitable molecular weight standard was selected as the Marker. The protein samples were mixed with 5× loading buffer and denatured by boiling at 95°C for 10 minutes. 20–30 μL of protein sample was loaded per well, and electrophoresis was performed at an appropriate voltage and time. The PVDF membrane was wetted in methanol and then transferred to the transfer buffer. The gel and membrane were sandwiched between two pieces of filter paper, removing any air bubbles, and the transfer was performed using appropriate parameters in the transfer apparatus. After transfer, the membrane was blocked with blocking solution (5% milk) for 1 hour to prevent non-specific binding. The membrane was incubated with diluted primary antibody overnight at 4°C, washed 3 times, then incubated with diluted secondary antibody for 1 hour at room temperature, and washed 3 times again. Finally, the membrane was treated with a chromogenic reagent and the signal was observed. ImageJ was used to process the images. β-Actin was used as an internal reference protein to calculate the relative expression levels of E-Cadherin, N-Cadherin, and Vimentin. All experimental results were validated through three repetitions.

### Transcription factor regulation analysis

Epithelial cells from pCRC and LM tissues were extracted and merged for transcription factor regulation analysis. Using the SCENIC package (v1.3.1) [35] in R and the GENIE algorithm, a co-expression network was constructed to predict transcription factor-target gene network modules (Regulons). The activity of Regulons was scored using AUCell, and specifically active Regulons were identified based on the area under the curve (AUC) values. Highly active Regulons were selected based on the AUC value, and EMT-related genes from the EMTome database [36] were screened to construct a ligand/receptor signal pair - Regulons network regulating CRC’s transition to EMT via SMCs and TEXs.

### Weighted co-expression network analysis (WGCNA)

SMCs from myeloid cells in pCRC and LM tissues were extracted and merged for WGCNA using the WGCNA package (v1.72-1) [37, 38] in R. Initially, gene modules were identified through the dynamic tree-cutting algorithm. Module eigengenes (MEs) were calculated for modules, and hierarchical clustering was performed on modules to obtain final gene modules. Subsequently, a correlation heatmap between modules and tissue sources was drawn to identify gene modules related to LM and obtain MEs for further analysis.

### Machine learning algorithm constructs prognostic risk model

Differential expression analysis was conducted on the merged SMCs to obtain DEGs. The intersection of DEGs with MEs of the turquoise module in WGCNA was used to obtain senescence-related genes (SRGs). To construct a prognostic risk model, 12 machine learning algorithms (Lasso, Ridge, Enet, Stepglm, SVM, glmBoost, LDA, plsRglm, RandomForest, GBM, XGBoost, NaiveBayes) were selected. In the framework of cross-validation, one algorithm was used for variable selection, while another algorithm was employed to construct a classification prediction model. The AUC under the ROC curve of the model combination in the dataset (including the training set and validation set) was calculated. The data for constructing the prognostic risk model were divided into a training set (TCGA-COAD dataset, https://portal.gdc.cancer.gov/repository) and a validation set (GEO dataset including GSE17536 [39], GSE17537 [39], GSE29621 [40], GSE38832 [41], and GSE39582 [42], https://www.ncbi.nlm.nih.gov/geo/). The best model was selected based on AUC. The formula for the prognostic risk model is as follows:


$$Risk\;Score=\sum_{i=1}^{n}Coe\;\int_{i}x_{i}$$

*Coe* ∫_*i*_ represents the risk coefficient, and *x_i_* denotes the gene expression level. Samples are categorized into high-risk and low-risk groups based on the median risk score. Kaplan-Meier survival curves are employed to visualize overall survival (OS) for all samples, both the training and validation sets.

### Data availability statement

The single-cell RNA sequencing (scRNA-Seq) data generated and analysed during the current study are available in the Gene Expression Omnibus (GEO) database, GSE178318 (http://www.ncbi.nlm.nih.gov/geo/query/acc.cgi?acc=GSE178318) and GSE205506 (https://www.ncbi.nlm.nih.gov/geo/query/acc.cgi?acc=GSE205506). The bulk RNA-Seq data generated and analysed during the current study are obtained from TCGA-COAD dataset (https://portal.gdc.cancer.gov/repository), GSE17536 (https://www.ncbi.nlm.nih.gov/geo/query/acc.cgi?acc=GSE17536), GSE17537 (https://www.ncbi.nlm.nih.gov/geo/query/acc.cgi?acc=GSE17537), GSE29621 (https://www.ncbi.nlm.nih.gov/geo/query/acc.cgi?acc=GSE29621), GSE38832 (https://www.ncbi.nlm.nih.gov/geo/query/acc.cgi?acc=GSE38832), and GSE39582 (https://www.ncbi.nlm.nih.gov/geo/query/acc.cgi?acc=GSE39582). The *in vitro* data are available upon reasonable request from the corresponding author, or in the ScienceDB database website https://www.scidb.cn/en/s/vyEBF3.

## RESULTS

### Data preprocessing and dimensionality reduction clustering

The scRNA-Seq data for this study were obtained from publicly available dataset. CRC tissues were collected from 16 patients, with matched pCRC and LM tissues. Quality control was performed based on three data features: the number of RNA features (nFeature), total gene expression within cells (nCount), and the proportion of mitochondrial genes (percent.mt). The quality control criteria were defined as follows: nCount between 1000 and 10000, nFeature between 250 and 4000, and percent.mt less than 15%. Initially, quality control was applied to the scRNA-Seq data from pCRC, and the data characteristics before and after quality control are presented in Supplementary Figure 1A, 1B. Following quality control, a total of 73,139 cells were retained. Subsequently, the data underwent SC-Transform normalization and harmony sample integration to correct batch effects. PCA was employed for dimensionality reduction clustering, and the optimal resolution of 0.9 was selected based on the clustree dendrogram, resulting in 36 clusters (Supplementary Figure 1C). Finally, UMAP was employed to visualize the results of dimensionality reduction clustering (Supplementary Figure 1D), and differential expression analysis was conducted using the Wilcoxon rank-sum test, with the top 5 DEGs in each cluster presented in a heatmap (Supplementary Figure 1E). The same preprocessing steps were replicated for the scRNA-Seq data of LM-CRC. Data characteristics before and after quality control are displayed in Supplementary Figure 2A, 2B, with 80,888 cells remaining after quality control. Batch effect correction was subsequently performed, and an appropriate resolution of 1.0 was chosen (Supplementary Figure 2C). PCA-based dimensionality reduction clustering yielded 29 clusters. UMAP was utilized to visualize the results (Supplementary Figure 2D), and the top 5 DEGs in each cluster were identified (Supplementary Figure 2E).

### Cell annotation

**Figure 1:**
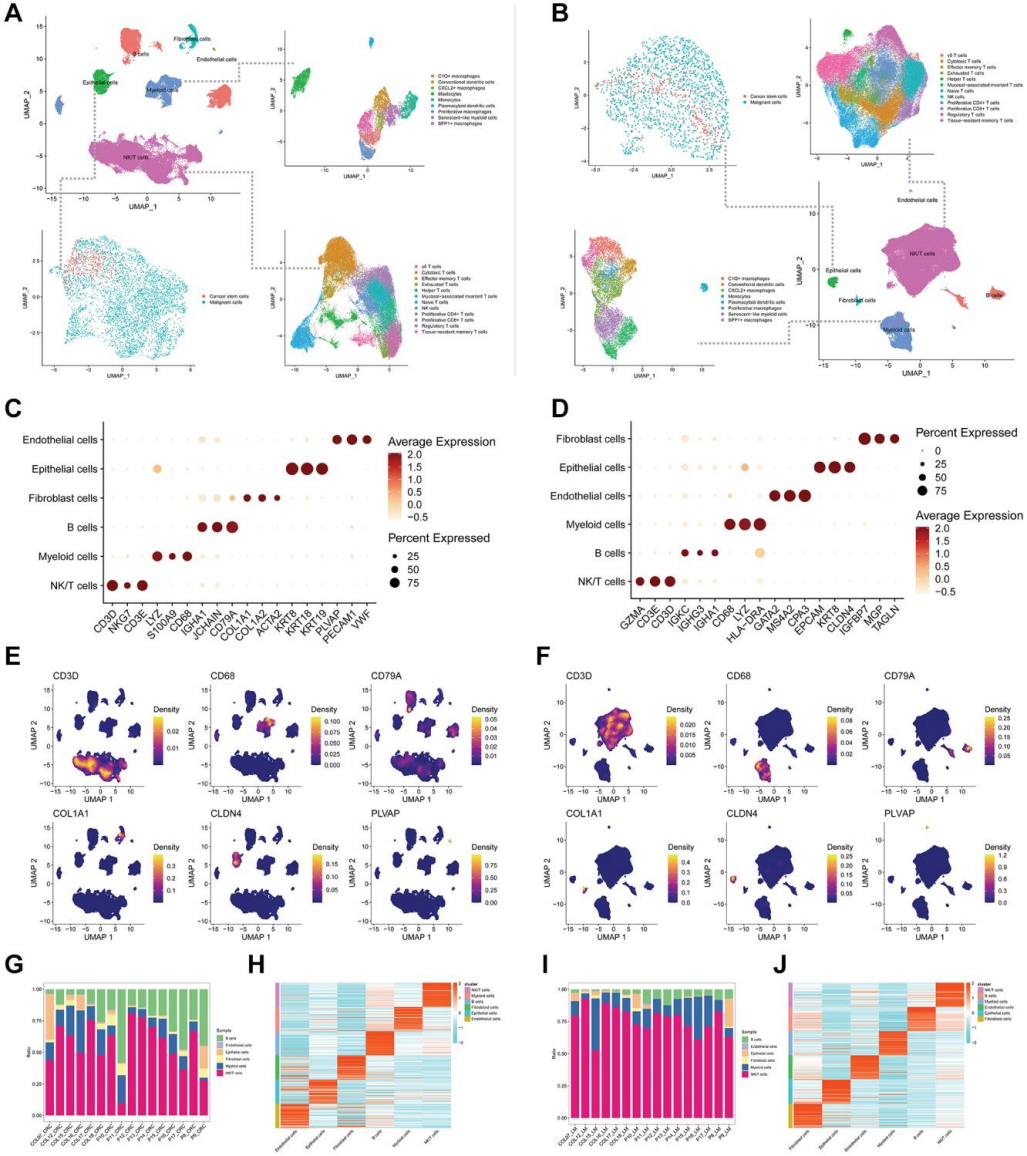
**Cell annotations and features of colorectal cancer tissues from primary and liver metastasis.** (**A**, **B**) UMAP-based dimensionality reduction clustering plots depict the annotation results of main cell types in primary (**A**) and liver metastasis (**B**) colorectal cancer tissues, along with further subannotations for epithelial cells, myeloid cells, and NK/T cells. (**C**, **D**) Bubble plots illustrate the expression levels of the top 3 markers in main cell types of primary (**C**) and liver metastasis (**D**) colorectal cancer tissues. (**E**, **F**) UMAP-based dimensionality reduction is employed to illustrate the density features of the top 1 marker in main cell types of primary (**E**) and liver metastasis (**F**) colorectal cancer tissues. (**G**) A proportion plot displays the distribution of primary cell types across 16 different primary colorectal cancer tissues. (**H**) A heatmap presents the expression levels of the top 50 differentially expressed genes in the main cell types of primary colorectal cancer tissues. (**I**) A proportion plot depicts the distribution of main cell types in 16 different liver metastasis colorectal cancer tissues. (**J**) A heatmap displays the expression levels of the top 50 differentially expressed genes in the main cell types of liver metastasis colorectal cancer tissues.

In this study, we performed cell annotation to categorize the main cell types found in pCRC (Figure 1A) and LM (Figure 1B) into six distinct groups, which include B cells, endothelial cells, fibroblast cells, epithelial cells, myeloid cells, and NK/T cells. Notably, our analysis of UMAP dimensionality reduction clustering plots for pCRC and LM demonstrated the clear demarcation of these six main cell types. However, it is of particular interest that within pCRC’s Myeloid cell population, two outlier clusters emerged, with one cluster closely associated with B cells. Upon further subtype annotation, these two clusters were identified as Mastocytes and plasma cell-like dendritic cells (Figure 1A). In contrast, the corresponding LM exhibited only one outlier cluster identified as plasma cell-like dendritic cells, with the Mastocytes subtype absent (Figure 1B). Subsequently, we characterized marker features for the main cell types present in pCRC and LM. The marker features for pCRC (Figure 1C) encompass B cells (IGHA1, JCHAIN, CD79A), endothelial cells (PLVAP, PECAM1, VWF), fibroblast cells (COL1A1, COL1A2, ACTA2), epithelial cells (KRT8, KRT18, KRT19), myeloid cells (LYZ, S100A9, CD68), and NK/T cells (CD3D, NKG7, CD3E), aligning with prevailing literature. It is worth mentioning that LM’s marker features exhibit slight divergence from those in pCRC (Figure 1D), especially in the case of endothelial cells (GATA2, MS4A2, CPA3) and fibroblast cells (IGFBP7, MGP, TAGLN), indicating a noteworthy distinction in the stromal cell composition between LM and pCRC. Furthermore, we depicted the expression patterns of primary cell type markers using density maps generated via UMAP dimensionality reduction (Figure 1E, 1F). Cell proportion plots (Figure 1G, 1I) reveal a higher proportion of epithelial cells and B cells in pCRC as compared to LM, while LM exhibits a significantly elevated proportion of NK/T cells. Detailed expression profiles of the top 50 DEGs for the six main cell types are presented in Figure 1H, 1J.

Moreover, we conducted dimensionality reduction clustering for epithelial cells, myeloid cells, and NK/T cells (Supplementary Figure 3A–3F), followed by the annotation of cell subtypes (Figure 1A, 1B). We designated epithelial cells as malignant cells and CSCs, identified SMCs within the Myeloid cell population, and pinpointed TEXs in the NK/T cell type. The distribution of cell subtypes can be observed in detail in Supplementary Figure 4A–4F. Notably, in LM’s myeloid cells, a substantial proportion of Mastocytes, present in pCRC, was not detected in the LM tissue. Additionally, the proportion of SMCs in LM was significantly higher than in pCRC (Supplementary Figure 4B, 4E). Within NK/T cell subtypes, pCRC exhibited a notably higher proportion of cytotoxic T cells compared to LM, whereas LM showed a higher proportion of naive T cells and TEXs (Supplementary Figure 4C, 4F).

**Figure 2:**
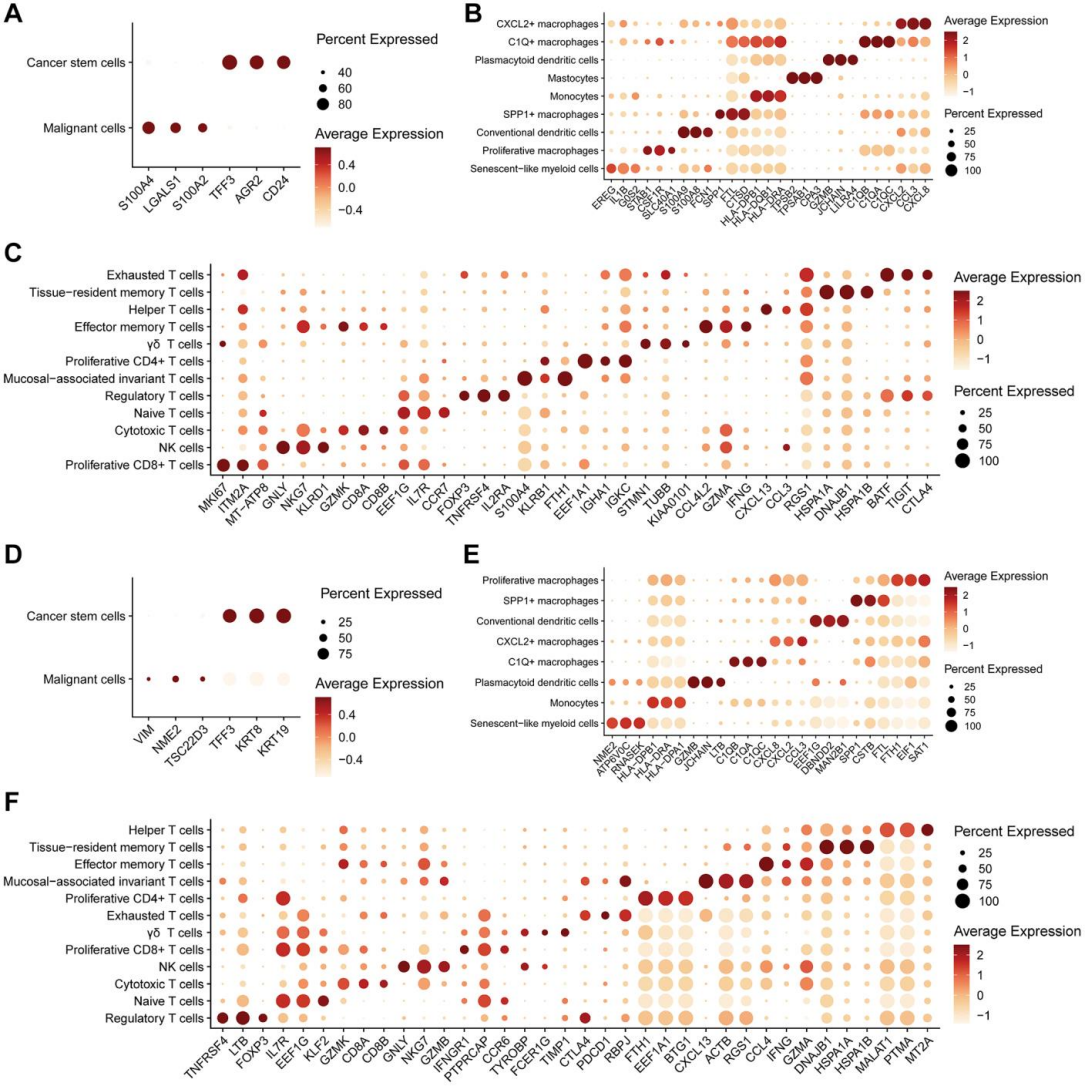
**Expression levels of markers in different cell subtypes.** (**A**–**C**) Bubble plots illustrate the expression levels of the top 3 markers in subtypes of epithelial cells (**A**), myeloid cells (**B**), and NK/T cells (**C**) in primary colorectal cancer tissues. (**D**–**F**) Bubble plots depict the expression levels of the top 3 markers in subtypes of epithelial cells (**D**), myeloid cells (**E**), and NK/T cells (**F**) in liver metastasis colorectal cancer tissues.

Through differential expression analysis, we identified distinctive markers for all cell subtypes within the three main cell types mentioned above (Figure 2A–2F). Our results underscore substantial heterogeneity between malignant tumor cells in pCRC and LM tissues. Notably, the markers for conventional tumor cells and CSCs exhibited notable differences, particularly with LM tissue displaying a more pronounced stem-like profile (Figure 2A, 2D). In myeloid cells, pCRC exhibited SMC markers, including EREG, IL1B, and G0S2 (Figure 2B), whereas LM displayed NME2, ATP6V0C, and RNASEK as markers (Figure 2E). Both sets of markers exhibited high expression of senescence-related genes, albeit with distinctive expression patterns. Furthermore, various cell subtypes in myeloid cells, such as CXCL2+ macrophages, C1Q+ macrophages, and SPP1+ macrophages, featured highly characteristic markers (Figure 2B, 2E), consistent across both pCRC and LM. However, it is essential to note that all Myeloid cell subtypes partially expressed genes from the HLA gene family, posing challenges in distinguishing specific monocyte subtypes, especially those expressing HLA-DPB1 and HLA-DRA. Within NK/T cells, TEXs were found in close proximity to regulatory T cells (Tregs), displaying elevated expression of immune inhibitory receptors. The markers for TEXs in pCRC (Figure 2C) and LM (Figure 2F) exhibited similar characteristics, marked by heightened expression of CTLA4, BATF, TIGIT, PDCD1, and other genes, all representing typical markers of T cell exhaustion.

### Cell trajectory analysis

**Figure 3:**
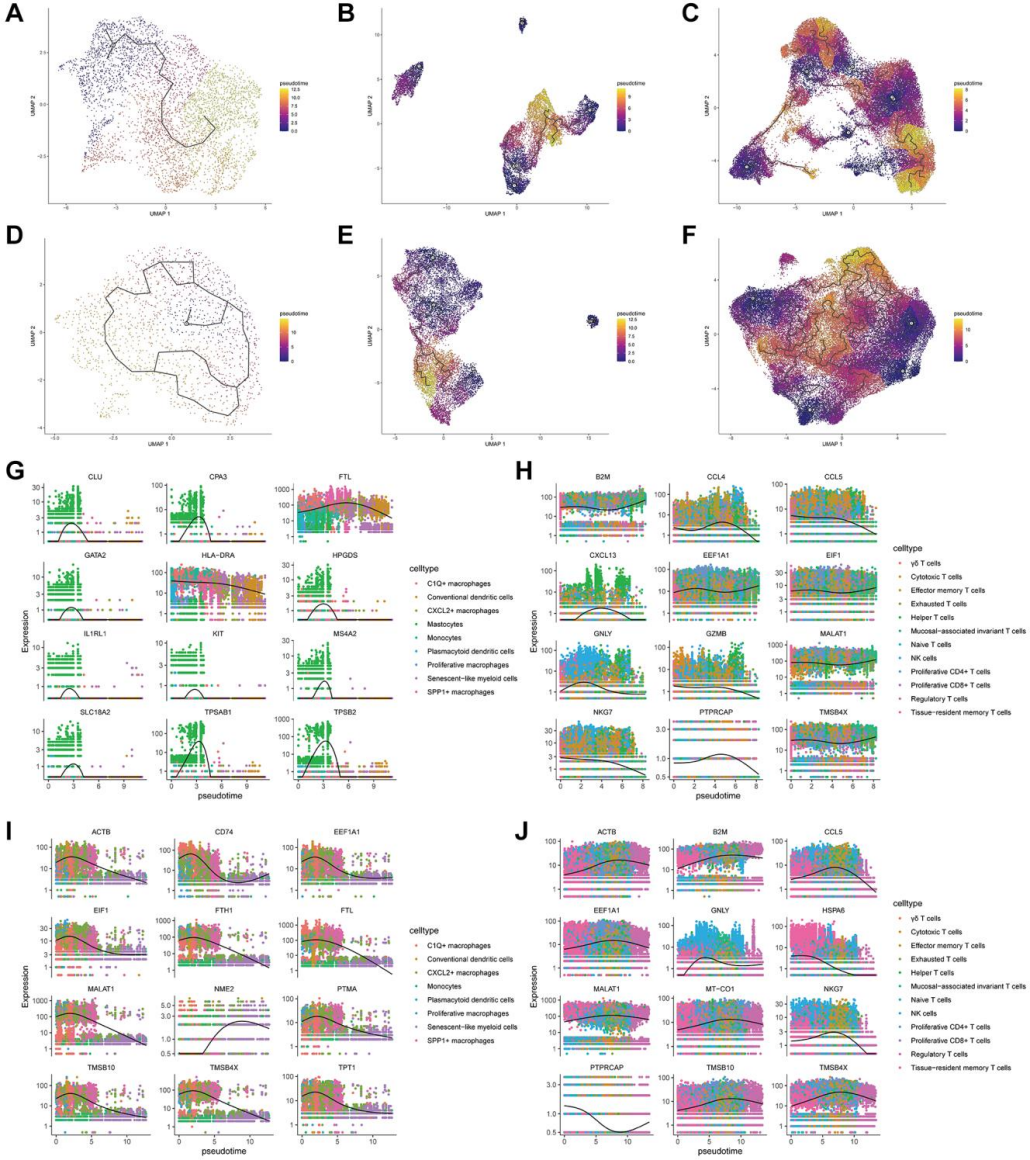
**Results of cell differentiation trajectory inference based on monocle3.** (**A**–**C**) Pseudotime cell differentiation trajectory plots for subtypes of epithelial cells (**A**), myeloid cells (**B**), and NK/T cells (**C**) in primary colorectal cancer tissues. (**D**–**F**) Pseudotime cell differentiation trajectory plots for subtypes of epithelial cells (**D**), myeloid cells (**E**), and NK/T cells (**F**) in liver metastasis colorectal cancer tissues. (**G**, **H**) Pseudotemporal expression trends of specific development-related genes in myeloid cells (**G**) and NK/T cells (**H**) in primary colorectal cancer tissues. (**I**, **J**) Pseudotemporal expression trends of particular development-related genes in myeloid cells (**I**) and NK/T cells (**J**) in liver metastasis colorectal cancer tissues.

In order to ascertain the developmental and differentiation trajectories of epithelial cells, myeloid cells, and NK/T cells, as well as to validate the precision of our cell subtype annotations, notably for CSCs, SMCs, and TEXs, we employed the monocle3 algorithm for cell trajectory analysis. Figure 3A–3C delineates the cell trajectories for epithelial cells, myeloid cells, and NK/T cells in pCRC, while Figure 3D–3F exhibit the cell trajectories for these same cell types in LM. Combining UMAP plots (Figure 1A, 1B) with cell trajectories reveals that in pCRC, CSCs are prominently positioned at the inception of the developmental trajectory (Figure 3A). However, in LM, CSCs are more broadly distributed in the early to mid-stage of development (Figure 3D). This divergence may be attributed to the relatively lower proportion of epithelial cells in LM, potentially resulting from an inadequate number of cells and overly dispersed clustering. SMCs in the cell trajectories of both pCRC and LM are situated toward the latter stages of pseudotime (Figure 3B, Figure 3E), displaying characteristics akin to terminal cells, which aligns with our cell subtype annotations. TEXs and Tregs are also closely situated along pseudotime, with both distributed toward the later stages of pseudotime (Figure 3C, 3F). Figure 3G–3J present genes whose expression undergoes significant changes along pseudotime (Moran’s I >0.8) for myeloid cells and NK/T cells. Notably, the presence of Mastocytes in pCRC’s Myeloid cell subtypes (Figure 3G, Figure 1A) resulted in substantial deviations in the calculation of Moran’s I, particularly for markers such as TPSAB1 and TPSAB2. In contrast, the development-related genes selected via Moran’s I values in LM align more closely with our subtype annotations, especially considering the absence of the Mastocytes subtype. Genes related to NK/T cell development in pCRC (Figure 3H) and LM (Figure 3J) exhibit similarities, encompassing cytotoxic markers (GNLY, NKG7), T cell proliferation markers (MALAT1), chemokines (CCL5), and Treg markers (TMSB4X).

### Enrichment analysis

**Figure 4:**
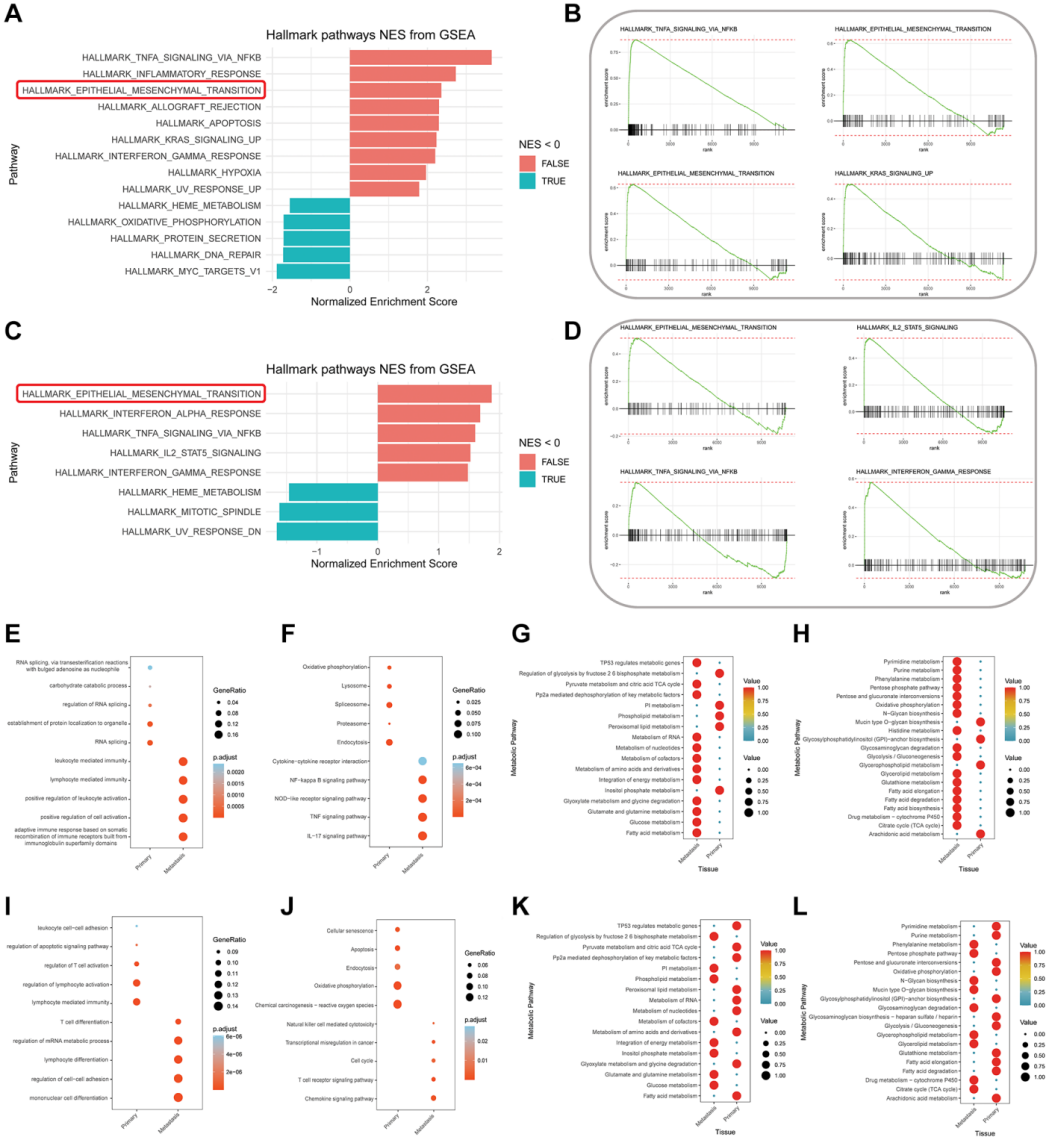
**Enrichment analysis results.** (**A**, **B**) Functional Gene Set Enrichment Analysis (fGSEA) for senescent-like myeloid cells (SMCs) subtypes from primary and liver metastasis colorectal cancer tissues, resulting in differential Hallmark signaling pathways (**A**) with selective GSEA plots (**B**). (**C**, **D**) fGSEA analysis for exhausted T cells (TEXs) subtypes from primary and liver metastasis colorectal cancer tissues, leading to differential Hallmark signaling pathways (**C**) with specific GSEA plots (**D**). (**E**–**H**) Enrichment analysis results for SMCs subtypes from primary and liver metastasis colorectal cancer tissues, based on Gene Ontology (GO) biological functions (**E**) and Kyoto Encyclopedia of Genes and Genomes (KEGG) signaling pathways (**F**), further analyzed for metabolic pathway enrichment through KEGG (**G**) and Reactome (**H**) pathways. (**I**–**L**) Enrichment analysis results for TEXs subtypes from primary and liver metastasis colorectal cancer tissues, based on GO biological functions (**I**) and KEGG signaling pathways (**J**), further analyzed for metabolic pathway enrichment through KEGG (**K**) and Reactome (**L**) pathways.

Based on the tissue of origin, we conducted comprehensive enrichment analyses encompassing biological functions, signaling pathways, and metabolic pathways for SMCs and TEXs subtypes. We prioritized GSEA analysis to explore whether SMCs and TEXs are associated with LM and to speculate on their pivotal mechanistic roles in promoting LM. The GSEA results for SMCs (Figure 4A) indicate significant enrichment in hallmark signaling pathways, including TNF-α signaling via NF-κB, EMT, Apoptosis, KRAS signaling UP, and IF-γ response, among others. Select GSEA enrichment plots for specific signaling pathways are displayed in Figure 4B. GSEA results for TEXs (Figure 4C) also unveil noteworthy enrichment in hallmark signaling pathways, encompassing EMT, IF-α response, TNF-α signaling via NF-κB, IL-2/STAT5 signaling, and IF-γ response, among others. Partial GSEA enrichment plots for specific signaling pathways are presented in Figure 4D. Notably, both SMCs and TEXs significantly enrich in TNF-α signaling via NF-κB and EMT signaling pathways, both intimately associated with malignant tumor metastasis, particularly EMT.

Furthermore, we conducted GO functional, KEGG signaling pathway, and metabolic pathway enrichment analyses, as illustrated in Figure 4E–4L. In the KEGG signaling pathway enrichment analysis for SMCs, we observed results similar to the GSEA analysis, with significant enrichment in the NK-κB signaling pathway and TNF signaling pathway in LM tissue (Figure 4E). Results of the signaling pathway enrichment analysis for TEXs (Figure 4J) predominantly indicate heightened immune pathway activities, encompassing cytotoxicity and chemotaxis, alongside cell cycle and transcription factor dysregulation in LM. The metabolic activity analysis suggests that SMCs may be involved in promoting glucose metabolism (glycolysis/gluconeogenesis and pentose phosphate pathway), lipid metabolism (fatty acid synthesis and degradation), nucleotide metabolism (purine and pyrimidine), and drug metabolism, in addition to participating in glycosylation (Figure 4G, 4H). These processes are closely related to tumor proliferation and drug resistance. Similar results were observed in the metabolic activity analysis for TEXs (Figure 4K, 4L), indicating their involvement in LM tissue’s glucose metabolism, lipid metabolism, drug metabolism, and glycosylation. Further in-depth research is warranted.

### Cell communication analysis

**Figure 5:**
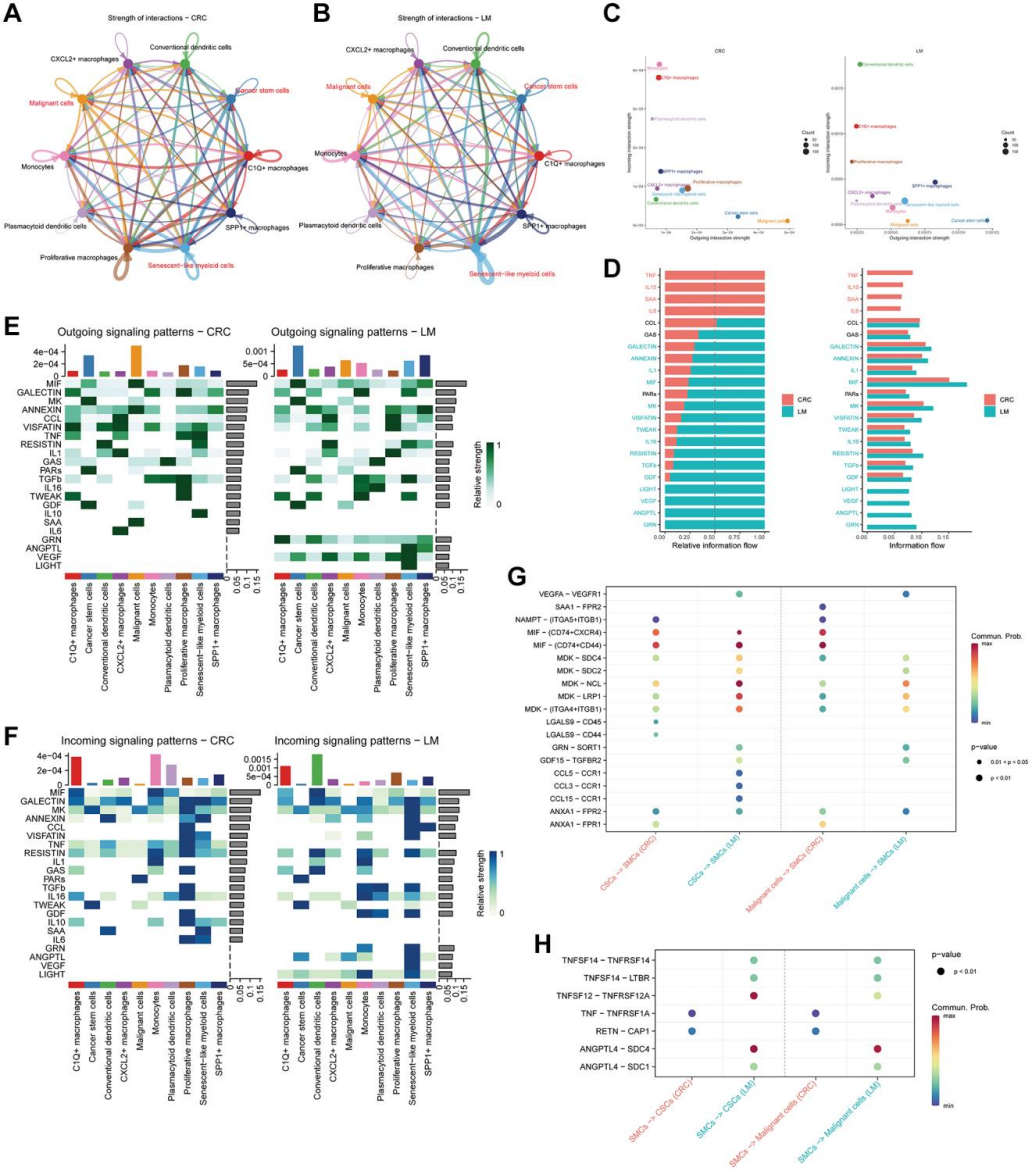
**Cell communication in different myeloid cell subtypes.** (**A**, **B**) Depiction of cell communication networks among distinct myeloid cell subtypes in primary (**A**) and liver metastasis (**B**) colorectal cancer tissues. (**C**) Utilization of bubble plots to visualize the intensity of cell communication outgoing and incoming signals. (**D**) Comparative analysis of signal strengths emanating from different secreted factors. (**E**, **F**) Heatmaps illustrating the intensity of various outgoing (**E**) and incoming (**F**) signal patterns within myeloid cell subtypes. (**G**) Presentation of bubble plots showcasing the signal strength of ligand-receptor pairs during interactions between malignant cells and tumor stem cells with myeloid cell subtypes. (**H**) Display of bubble plots illustrating the signal strength of ligand-receptor pairs during interactions of myeloid cell subtypes with malignant cells and tumor stem cells.

Our study entailed an in-depth cell communication analysis focused on myeloid cells and NK/T cells, with specific attention to elucidating the crosstalk between SMCs and TEXs and tumor cells originating from distinct tissues. Our analysis began by contrasting the patterns of cell communication among various myeloid cell subtypes in pCRC (Figure 5A) and LM (Figure 5B). This comparison, coupled with bubble plots demonstrating the strength of both outgoing and incoming signals (Figure 5C), revealed a conspicuous trend: SMCs and CSCs in LM exhibited a substantial amplification in outgoing signal strength compared to their pCRC counterparts. Notably, SMCs demonstrated a particularly notable increase in outgoing signal strength directed toward CSCs. Delving deeper into the overall signaling patterns within pCRC and LM (Figure 5D), we discerned that the dominant signaling pattern in pCRC corresponded to the SASP phenotype, characterized by robust signaling of senescence-related secretory factors, such as IL-10 and IL-6. In contrast, LM exhibited heightened signaling in various secretory factors, including MIF, LIGHT (TNFSF14), VEGF, ANGPTL, and GNR. Subsequent to this, our analysis delved into the outgoing (Figure 5E) and incoming (Figure 5F) signal strength of secretory factors across a spectrum of cell subtypes. This comprehensive evaluation disclosed that cell subtypes within LM collectively demonstrated enhanced outgoing signals for secretory factors like MIF, VEGF, ANGPTL, and GNR. A noteworthy observation was the significantly elevated outgoing signals from SMCs, especially concerning VEGF and ANGPTL. These findings bear relevance to vascular endothelial cell survival, maturation, and migration. Finally, we analyzed the differences in signal intensity among various ligand-receptor combinations in both pCRC and LM. Our analysis revealed that in regulating signal transmission from tumor cells to smooth muscle cells (SMCs) - as shown in Figure 5G - the signal intensity of VEGFA-VEGFR1, MDK-SDC1/SDC4, MDK-LRP1, MDK-(ITGA4+ITGB1), GNR-SORT1, and GDF15-TGFBR2 exhibits higher levels in LM, whereas LGALS9-CD44/CD45 predominates in pCRC. Concerning the regulation of signal transmission from SMCs to tumor cells, as depicted in Figure 5H, the signal intensity of TNFSF14-TNFRSF14/LTBR, TNFSF12-TNFRSF12A, and ANGPTL4-SDC1/SDC4 demonstrates higher levels in LM, whereas TNF-TNFRSF1A and RETN-CAP1 are predominant in pCRC. The signaling pathway involving ANGPTL4-SDC1/SDC4 is closely associated with both cell adhesion and vascular development, indicating that SMCs may contribute to the development of LM through the ANGPTL4-SDC1/SDC4 signaling pathway.

**Figure 6:**
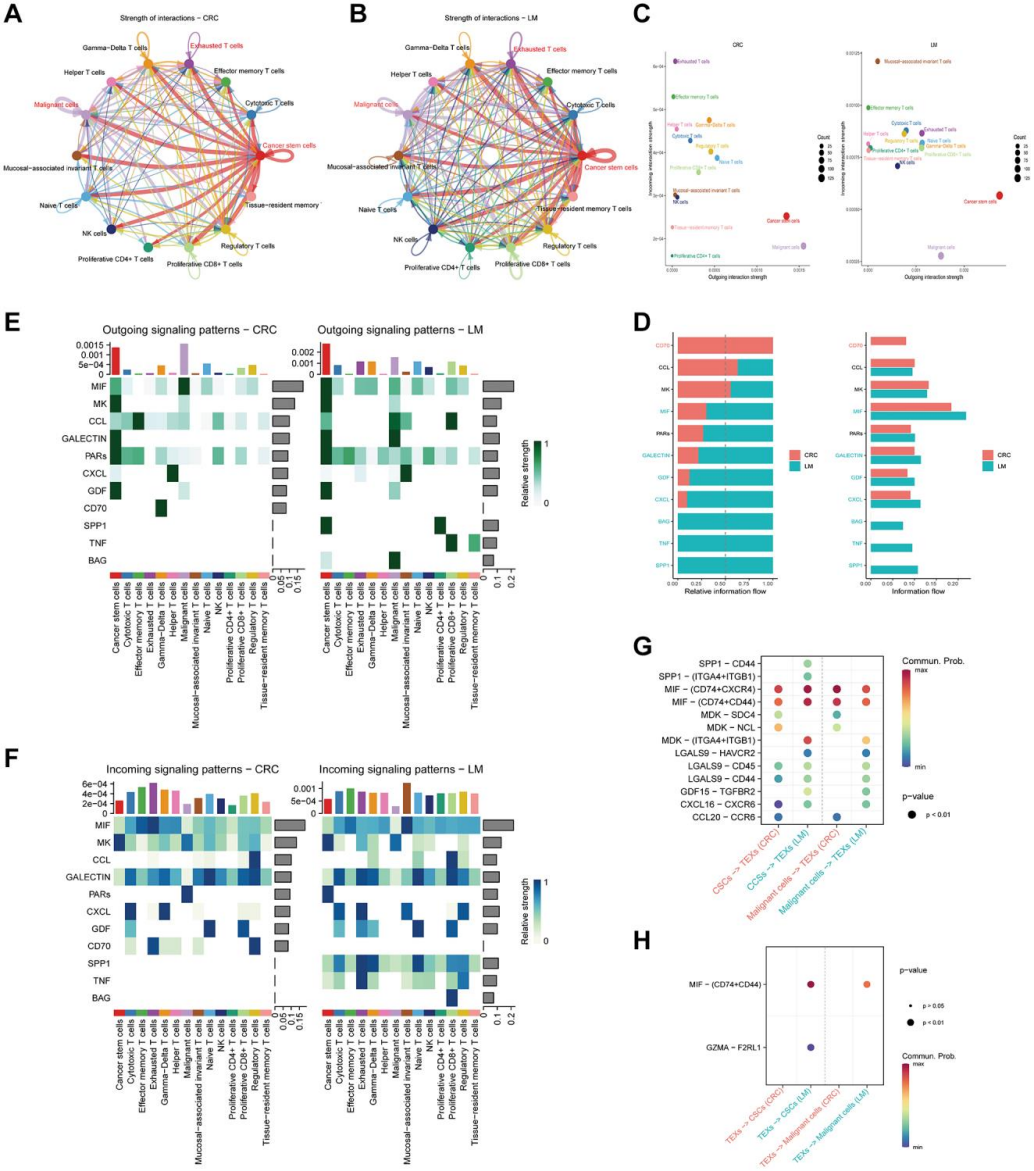
**Cell communication in various NK/T cell subtypes.** (**A**, **B**) Representation of cell communication networks among different NK/T cell subtypes in primary (**A**) and liver metastasis (**B**) colorectal cancer tissues. (**C**) Application of bubble plots to depict the strength of cell communication outgoing and incoming signals. (**D**) Comparative assessment of signal strength from distinct secreted factors. (**E**, **F**) Heatmaps delineating the intensity of diverse outgoing (**E**) and incoming (**F**) signal patterns within NK/T cell subtypes. (**G**) Visualization of bubble plots highlighting the signal strength of ligand-receptor pairs involved in interactions between malignant cells and tumor stem cells with TEXs subtypes. (**H**) Exhibition of bubble plots displaying the signal strength of ligand-receptor pairs during the interactions of TEXs subtypes with malignant cells and tumor stem cells.

Our investigation then extended to the creation of a cell communication network for NK/T cells. It is evident that the overall interaction strength in pCRC (Figure 6A) was comparatively lower than that in LM (Figure 6B). Bubble plots, detailing the strength of outgoing and incoming signals, are depicted in Figure 6C. These representations underscored a conspicuous difference: TEXs and CSCs in LM exhibited notably heightened outgoing and incoming signal strengths in comparison to their pCRC counterparts, with TEXs displaying a particularly prominent surge in outgoing signal strength. A broader examination of the overarching signaling patterns (Figure 6D) revealed that pCRC exhibited more robust signaling in CD70, whereas LM displayed heightened signaling in several secretory factors, including MIF, BAG, TNF, and SPP1. Subsequent scrutiny of the outgoing (Figure 6E) and incoming (Figure 6F) signal strength of secretory factors across diverse cell subtypes unveiled that, relative to pCRC, LM displayed heightened outgoing signal strength for MIF in TEXs, and an increased outgoing of SPP1, GALECTIN, and BAG in CSCs and malignant cells. In terms of incoming signals, TEXs in LM demonstrated a higher signal strength for TNF, SPP1, and CXCL in comparison to their pCRC counterparts, while CSCs displayed an elevated PARs signaling strength. Finally, bubble plots (Figure 6G) portraying the differences in ligand-receptor signal pair strengths between pCRC and LM demonstrated variations in the intensity of signal regulation by tumor cells in TEXs. Specifically, when compared to pCRC, LM exhibited higher signal strength in pairings such as SPP1-CD44/(ITGA4+ITGB1), MDK-(ITGA4+ITGB1), and GDF15-TGFBR2, while signal strength in MDK-SDC4/NCL pairings was lower. In the context of signal regulation by TEXs in tumor cells (Figure 6H), our findings pointed to MIF-(CD74+CD44) as a significantly more potent regulatory signal, playing a pivotal role in driving malignant tumors.

### *In vitro* experimental results

**Figure 7:**
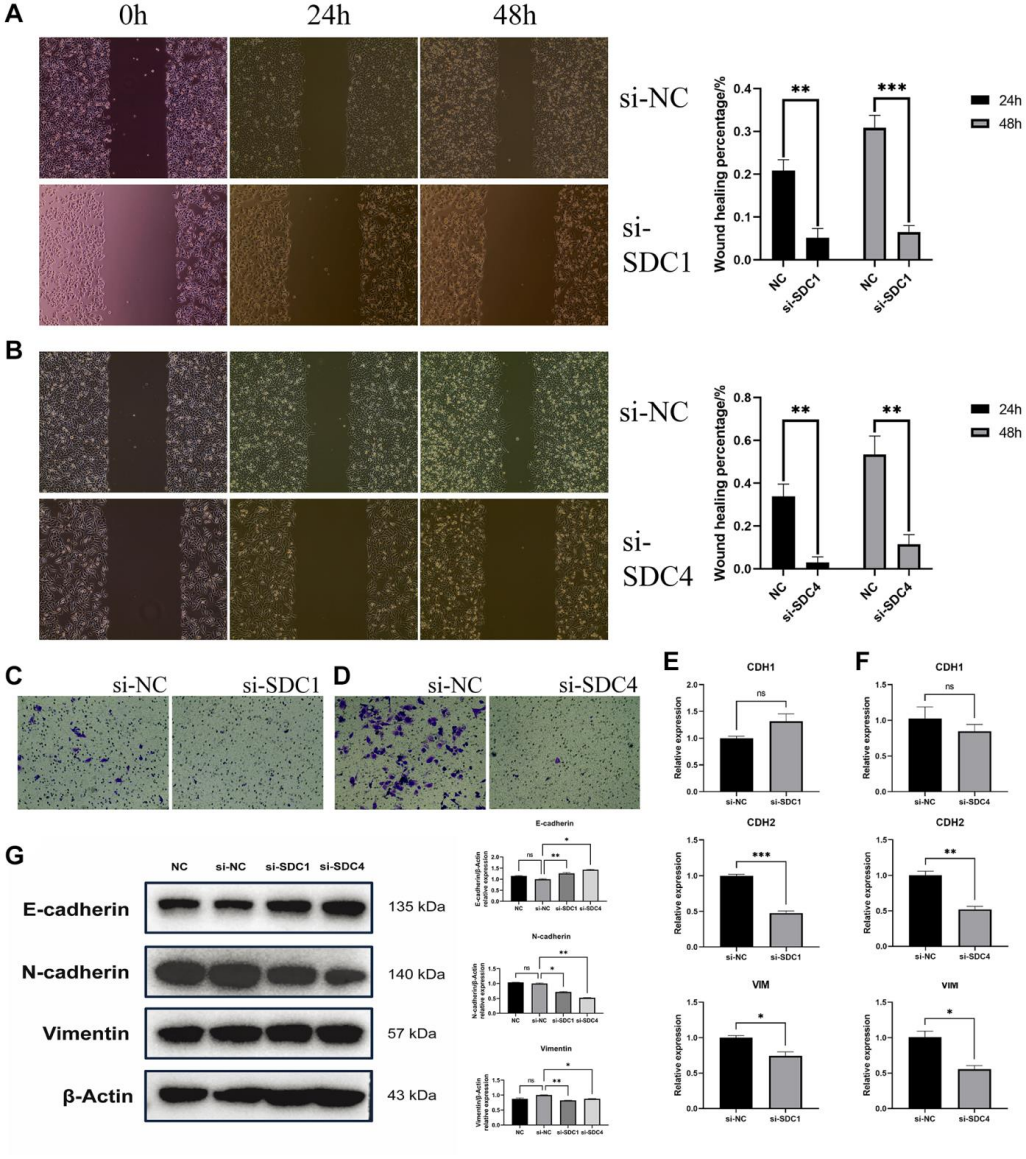
***In vitro* experimental results.** (**A**) Evaluation of scratch tests conducted at 24 h and 48 h following SDC1 knockdown, relative to the negative control group, with subsequent determination of wound healing percentage. (**B**) Assessment of scratch tests performed at 24 h and 48 h following SDC4 knockdown, relative to the negative control group, with subsequent determination of wound healing percentage. (**C**) Analysis of Transwell migration experiments following SDC1 knockdown, relative to the negative control group. (**D**) Examination of Transwell migration experiments following SDC4 knockdown, relative to the negative control group. (**E**) Contrasting the mRNA relative expression levels of CDH1, CDH2, and VIM following SDC1 knockdown with those of the negative control group. (**F**) Contrasting the mRNA relative expression levels of CDH1, CDH2, and VIM following SDC4 knockdown with those of the negative control group. (**G**) Contrasting the protein relative expression levels of E-cadherin, N-cadherin, and Vimentin following SDC1 or SDC4 knockdown with those of the negative control group. (^*^P < 0.05, ^**^P < 0.01, ^***^P < 0.001).

The wound healing test results indicated that, in comparison to the si-NC group, the si-SDC1 group exhibited reduced cell migration capability in HCT116 cells at both 24 h (*P* = 0.0013) and 48 h (*P* = 0.0002) following SDC1 knockdown (Figure 7A). Similarly, observations in the si-SDC4 group at 24 h (*P* = 0.0011) and 48 h (*P* = 0.0017) confirmed decreased cell migration ability in HCT116 cells upon SDC4 knockdown (Figure 7B). Subsequent Transwell migration experiments illustrated that, after SDC1 (Figure 7C) or SDC4 (Figure 7D) knockdown, HCT116 cells displayed diminished migratory capacity, particularly evident after SDC4 knockdown, where a marked reduction in cell migration ability was evident.

RT-qPCR results revealed that upon SDC1 knockdown, although CDH1 expression in the si-SDC1 group (Figure 7E) did not significantly differ from the si-NC group (*P* = 0.0952), the expression levels of CDH2 (*P* = 0.0001) and VIM (*P* = 0.0163) were significantly lower than those in the si-NC group. Similarly, CDH1 expression in the si-SDC4 group (Figure 7F) showed no significant difference from the si-NC group (*P* = 0.2632), but CDH2 (*P* = 0.0024) and VIM (*P* = 0.0104) expression levels were significantly lower.

Western blot (Figure 7G) analysis revealed that, compared to si-NC group, the expression levels of E-Cadherin (*P*_si-SDC1_ = 0.0076, *P*_si-SDC4_ = 0.0161) were markedly increased following SDC1/SDC4 knockdown. However, the expression levels of N-Cadherin (*P*_si-SDC1_ = 0.0219, *P*_si-SDC4_ = 0.0025) and Vimentin (*P*_si-SDC1_ = 0.0071, *P*_si-SDC4_ = 0.0241) were markedly decreased following SDC1/SDC4 knockdown. These findings are in agreement with the results obtained from RT-qPCR experiments. In conclusion, the *in vitro* experimental findings suggest that the knockdown of both SDC1 and SDC4 can suppress the migratory capability of HCT116 cells, and these mechanisms may be associated with inhibiting the occurrence of EMT.

### Transcription factor regulation analysis

**Figure 8:**
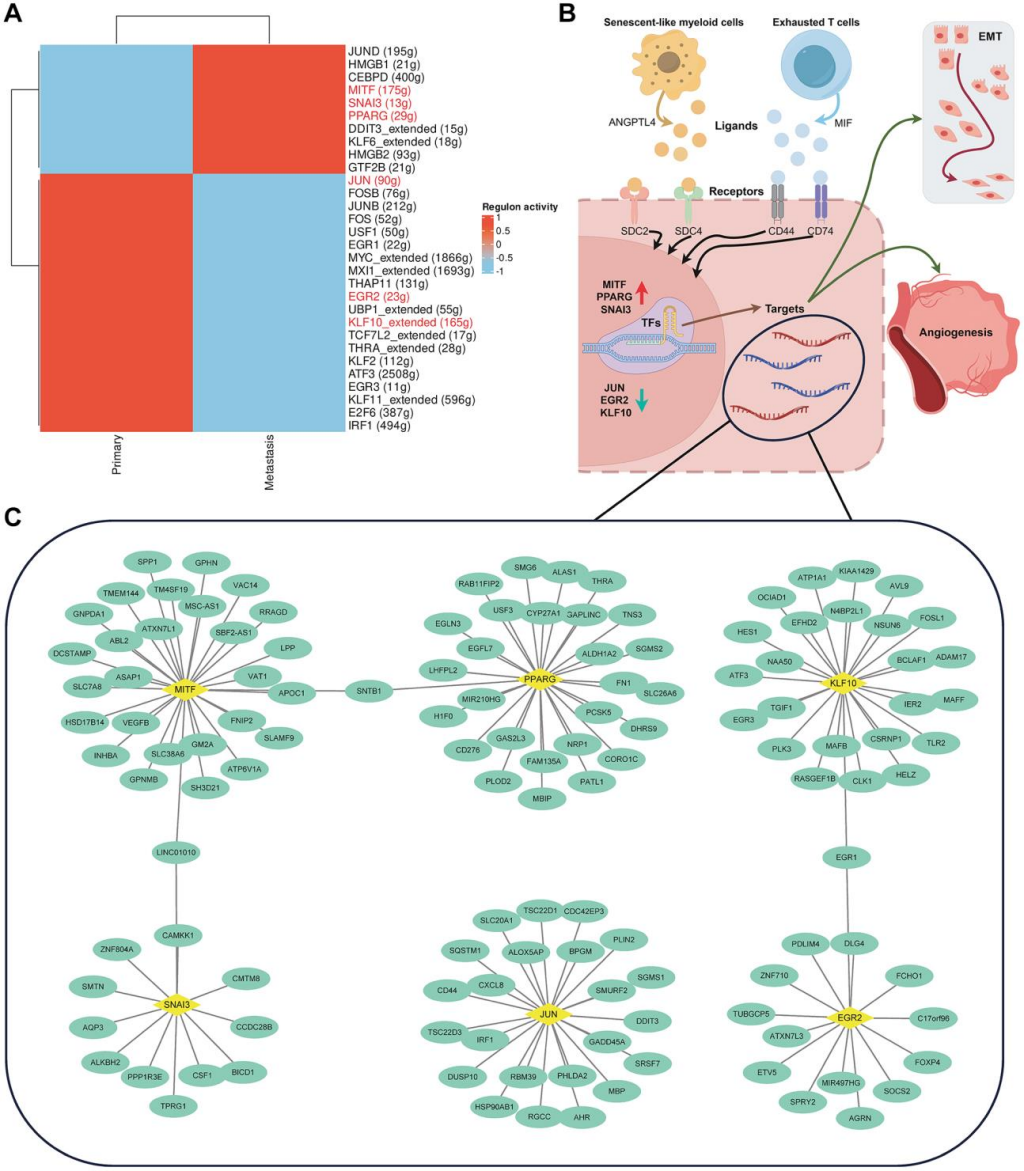
**Analysis of transcription factor activity.** (**A**) Presentation of a heatmap illustrating variations in transcription factor activity between primary and liver metastasis colorectal cancer tissues. (**B**) Exploration of the molecular mechanisms by which aging and exhausted immune cells promote CRC progression and metastasis. (**C**) Detailed annotation of the transcription factor-target regulatory network.

For malignant tumor cells, we reconstructed transcription factor-gene regulatory networks using the SCENIC algorithm. By conducting an EMTome analysis of transcription factors, we identified those associated with EMT. The results indicated that, compared to pCRC, transcription factors MITF, SNAI3, and PPARG in LM exhibited specific high activity, whereas transcription factors JUN, ERG2, and KLF10 displayed low activity (Figure 8A). Additionally, HMGB1, HMGB2, and CEBPD exhibited elevated activity. Based on enrichment analysis, cell communication, and transcription factor regulation analysis, we hypothesize that senescent and exhausted immune cells promote EMT and angiogenesis in CRC, thereby contributing to the molecular mechanisms underlying tumor progression and metastasis (Figure 8B). The EMT-related Regulons network is illustrated in Figure 8C.

### WGCNA and prognostic risk model construction

**Figure 9:**
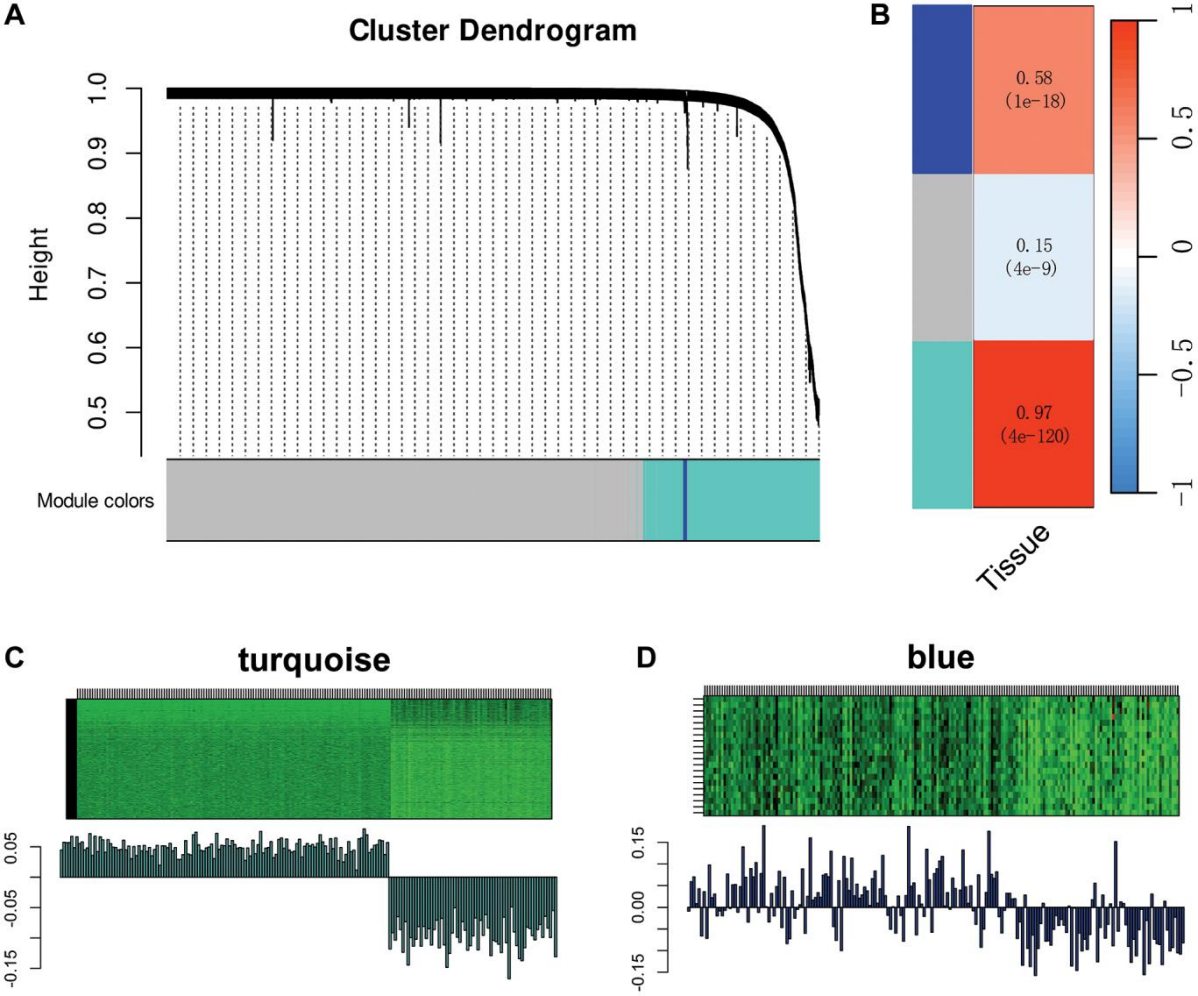
**Weighted co-expression network analysis (WGCNA) of SMCs subtypes.** (**A**) Depiction of a dendrogram demonstrating the clustering of different gene modules in WGCNA. (**B**) Examination of the correlation between gene module expression levels and the source of colorectal cancer tissues (primary/liver metastasis) for different gene modules (turquoise and blue). (**C**, **D**) Representation of gene expression levels for the turquoise module (**C**) and blue module (**D**) across all samples.

Through prior multi-omics analyses, we ascertained that SMCs might be closely associated with LM through mechanisms such as EMT and angiogenesis promotion. Therefore, we employed WGCNA to identify gene modules highly correlated with LM. Two gene modules, namely the turquoise module and the blue module, were identified through WGCNA (Figure 9A). Correlation analysis results (Figure 9B) indicated that both the turquoise and blue modules correlated with the tissue source of SMCs. By examining the heatmaps of MEs expression levels for both gene modules (Figure 9C, 9D), it became apparent that the turquoise module exhibited a highly significant correlation with the occurrence of LM.

**Figure 10:**
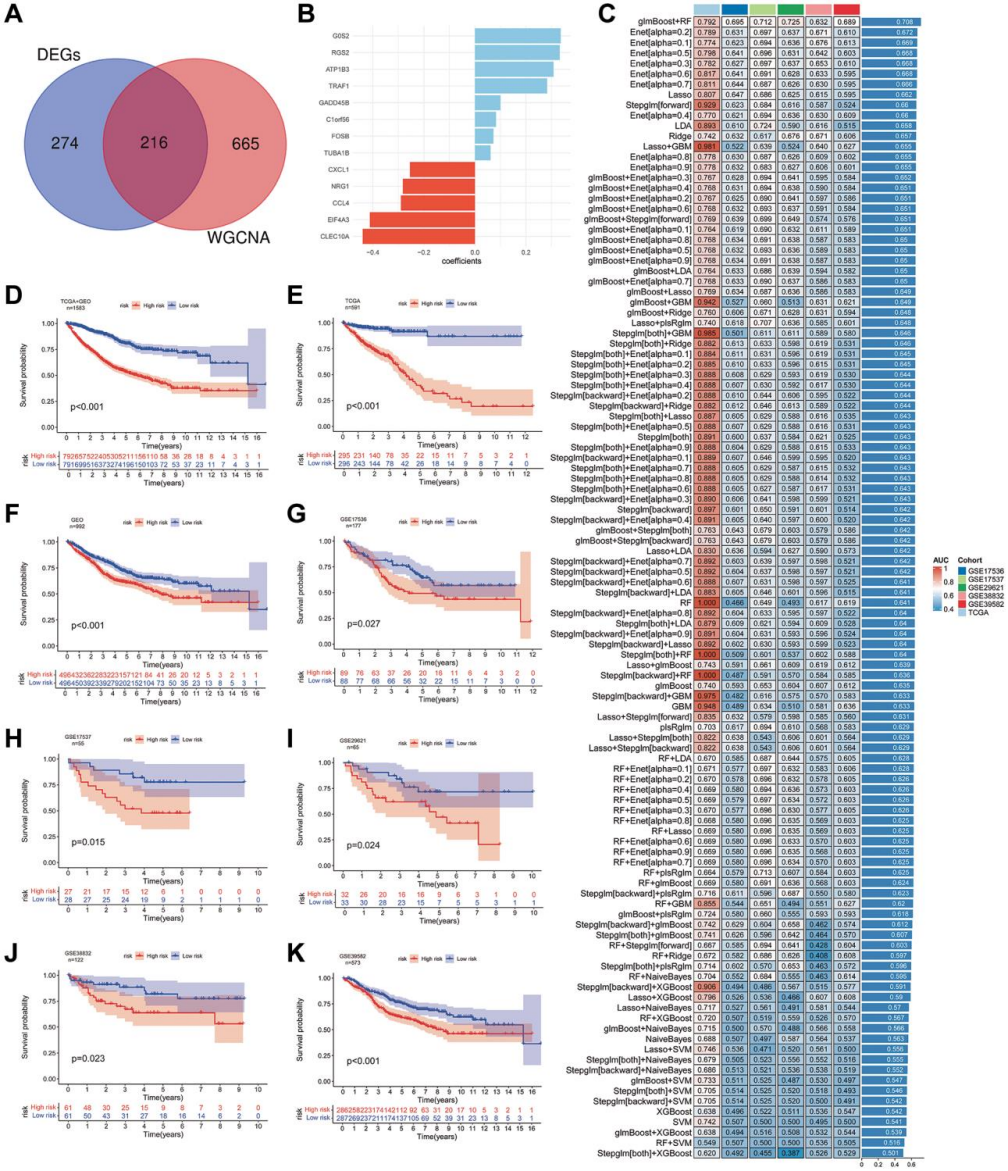
**Development of prognostic risk models using machine learning algorithms.** (**A**) Identification of the intersection between differentially expressed genes and genes from the turquoise module within subtypes of SMCs in primary and liver metastasis colorectal cancer tissues. (**B**, **C**) Construction of prognostic risk models employing 113 machine learning algorithms. The bar chart illustrates the relevant genes and their correlation coefficients (**B**), while the heatmap displays the AUC values for training and validation cohorts under various algorithms (**C**). (**D**–**F**) Depiction of Kaplan-Meier survival curves for high- and low-risk groups within all samples (**D**), training cohorts (**E**), and validation cohorts (**F**). (**G**–**K**) Display of Kaplan-Meier survival curves representing high- and low-risk groups in validation cohorts from GSE17536 (**G**), GSE17537 (**H**), GSE29621 (**I**), GSE38832 (**J**), and GSE39582 (**K**) samples.

To construct a prognostic risk model, we employed 12 machine learning algorithms for model development and validation through a combination of 113 models. Initially, we selected 216 SRGs by taking the intersection of 490 DEGs and 881 MEs in the turquoise module (Figure 10A). After constructing and validating machine learning models, we determined that a prognostic risk model combining the glmBoost and RandomForest algorithms yielded the best results (Figure 10B). The model consisted of 13 genes (G0S2, RGS2, ATP1B3, TRAF1, GADD45B, C1orf56, FOSB, TUBA1B, CXCL1, NRG1, CCL4, EIF4A3, CLEC10A). Evaluating the AUC of the training and validation datasets, we found that the prognostic risk model exhibited robust predictive capability for CRC’s OS (Figure 10C). Subsequently, we computed risk values for all samples and classified samples into high- and low-risk groups using the median risk value. Kaplan-Meier survival curves portrayed the OS of these groups. Across all samples (Figure 10D), the training dataset (Figure 10E), and the validation dataset (Figure 10F), we consistently observed that the low-risk group had a significantly higher OS compared to the high-risk group. Furthermore, the Kaplan-Meier survival curves for the five validation subsets (Figure 10G–10K) reaffirmed this conclusion.

## DISCUSSION

CRC represents a formidable malignant disease with a substantial impact on human health. One of the predominant challenges in current systemic treatment is to enhance the OS of patients with metastatic mCRC. Given the distinct attributes of the TME within CRC, existing targeted therapies and immunotherapies struggle to confer sustained survival benefits to the majority of mCRC patients. The principal hindrances to achieving these benefits are the restricted indications and the emergence of drug resistance. Hence, there exists an imperative need to uncover a breakthrough in our understanding of the molecular mechanisms that underlie the distant metastasis of CRC. Central to this discussion is the concept that immune cells manifesting features of senescence and exhaustion play a pivotal role in reshaping the TME and are intimately associated with the metastatic progression of malignant tumors. To delve into the nexus between senescent and exhausted immune cells and the onset of distant metastasis in CRC, our study harnessed scRNA-Seq data for the identification of these immune cell profiles. Employing a single-cell multi-omics approach, we meticulously probed the potential mechanisms by which SMCs and TEXs facilitate liver metastasis in the context of CRC. Furthermore, our research entailed the development of a prognostic risk model employing a suite of 12 machine learning algorithms to predict the correlation between genes associated with senescence and OS, thereby ushering in novel insights into the TME in the context of colorectal cancer liver metastasis.

In the realm of main cell type annotation, it is evident that the proportions of NK/T cells, myeloid cells, and B cells exhibit marked discrepancies between pCRC and LM. Within the NK/T cell subtypes, pCRC demonstrates a substantially heightened representation of cytotoxic T cells, while LM exhibits an augmented prevalence of naïve T cells and TEXs. This disparity implies a diminished cytotoxic influence within the TME of LM, supplanted by an inhibitory effect. Of particular interest is the prevalence of mastocytes in myeloid cell subtypes in pCRC, a feature not discerned in LM. Intriguingly, mastocytes have been historically underexplored in prior research, yet recent investigations underscore their significance in the context of CRC prognosis and therapy. Mast cells have a pivotal role in the progression of colorectal cancer through the release of granules containing angiogenic factors such as VEGF-A, CXCL8, MMP-9, and lymphangiogenic factors like VEGF-C and VEGF-D [43]. Furthermore, SMCs manifest a substantially higher proportion within myeloid cells in LM compared to pCRC. To summarize, the observed alterations in the ratios of cell subtypes within NK/T cells and myeloid cells not only unveil an inhibitory TME within LM but also intimate a close interplay between SMCs and TEXs in the initiation of LM. These findings furnish a preliminary basis for our subsequent in-depth inquiries.

Subsequently, we identified distinctive markers characteristic of cell subtypes and elucidated the pseudo-temporal sequence of these cell subtypes via differentiation trajectories. It emerged that both SMCs and TEXs exhibited noteworthy attributes in both pCRC and LM. In SMCs, aside from the conspicuous overexpression of markers associated with the SASP like IL1B and EREG [44, 45], there was a substantial upregulation of ATP6V0C, G0S2, and RNASEK. The extant body of research suggests that ATP6V0C [46] and G0S2 [47] can elicit autophagy and apoptosis in senescent cells, thereby facilitating the elimination of senescent cells [48, 49]. RNASEK, as a constituent of the ribonuclease family, expedites RNA degradation, thereby accelerating the clearance of senescent cells [50]. This peculiar marker expression profile in SMCs reflects the organism’s strategy for the removal of senescent cells, a phenomenon meriting further exploration given its relative novelty in prior research [51]. Comparatively, the markers characterizing TEXs closely adhere to existing research [52], marked by a consistent upregulation of immunosuppressive receptors such as CTLA4, BATF, TIGIT, and PDCD1, irrespective of the tissue source. Notably, the trajectory analysis of cell populations establishes a proximate relationship between TEXs and Tregs, emphasizing the significant role of TEXs in configuring an inhibitory TME.

To elucidate the prospective mechanisms through which SMCs and TEXs expedite the initiation of liver metastasis, our study harnessed predictive techniques encompassing enrichment analysis, cell communication, and transcription factor activity. It has been ascertained that Angiopoietin-like protein 4 (ANGPTL4) exerts influence on the progression and metastasis of CRC through various pathways [53]. Current research [54] suggests that ANGPTL4 primarily fuels tumor progression and metastasis through its C-terminal fragment. Notably, ANGPTL4 also functions as a crucial metabolic regulator [55], implicated in genetic variations relating to a broad spectrum of blood lipids and metabolites. Furthermore, it plays a role in regulating the expression of glucose transport proteins, thereby promoting glucose metabolism within CRC [56]. The outcomes of cell communication analysis elucidate that SMCs in LM regulate the behavior of CRC cells, including CSCs, through the secretion of ANGPTL4.

A selection of studies has indicated that SDC1 can suppress EMT in oral cancer cells. Conversely, SDC1 has been implicated in mediating EMT in prostate cancer. Nevertheless, SDC4, although less explored in the context of EMT, has been reported to positively regulate TGF-β1-induced EMT in lung adenocarcinoma cells. Notably, no research has thus far probed the association of SDC1 and SDC4 with EMT within the milieu of CRC. Consequently, our research ventured into experimental validation. The results conclusively demonstrate that the silencing of SDC1 and SDC4 enhances the expression of E-Cadherin (CDH1) while concurrently diminishing the presence of N-Cadherin (CDH2) and Vimentin (VIM) in HCT116 cells. This empirical verification affirms that both SDC1 and SDC4 play an integral role in promoting EMT in CRC cells, consequently propelling tumor progression and metastasis, a finding consistent with our earlier results derived from enrichment analysis. Furthermore, a subset of studies has revealed that tumor-associated macrophages can wield influence over the EMT program in CRC through the secretion of SASP molecules, such as IL-6, thereby intensifying CRC metastasis [57]. In the case of TEXs, cell communication analysis demonstrates that MIF-(CD74+CD44) exhibits substantially higher signal intensity in LM, and MIF is an upstream regulator of the EMT program. The upregulation of MIF is documented to attenuate E-Cadherin in tumor cells, consequently augmenting their proliferation, invasive properties, and migratory capacity *in vitro* [58, 59]. In summary, our postulation posits that SMCs and TEXs leverage the interaction between ligand-receptor pairs, such as ANGPTL4-SDC1/SDC4 and MIF-(CD74+CD44), in cell communication to activate downstream transcription factors, thereby participating in the regulation of the EMT program within the context of CRC and precipitating the emergence of LM.

In the subsequent phase of cell communication analysis, we embarked on a thorough analysis of transcription factor regulation. By selecting transcription factors linked to EMT, we determined that within LM, specific transcription factors, such as SNAI3, MITF, and PPARG, manifest distinctively high activity levels. Among these highly active transcription factors, members of the SNAIL family (SNAI1, SNAI2, SNAI3) emerge as quintessential regulators of EMT. They wield their influence by suppressing specific target genes, notably the E-Cadherin gene (CDH1), thereby underscoring their association with the adverse prognosis observed in metastatic cancer [60]. MITF, a pivotal regulatory element in melanocyte development and differentiation, plays a facilitating role in EMT within the extracellular matrix of melanoma cells [61]. The heightened expression of PPARG in intestinal epithelium similarly typifies its involvement in EMT regulation [62]. Notably, the augmented activity of HMGB1 within LM fosters angiogenesis [63], while the diminished activity of KLF10 serves as a suppressive regulator of EMT [64]. In conclusion, the TME of LM distinctly exhibits characteristics that promote EMT and angiogenesis.

In light of the cumulative findings derived from enrichment analysis, cell communication, cell trajectory, and transcription factor regulation, we posit that senescent and exhausted immune cells are instrumental in propelling EMT and angiogenesis, thereby culminating in tumor progression and metastasis. However, this study is by no means exhaustive. The definitive validation of the molecular mechanisms underpinning the role of senescent and exhausted immune cells in the promotion of LM necessitates further elaboration. Throughout the course of our analysis, a plethora of intriguing phenomena has surfaced, not least the intricate interplay between mastocytes, which predominate in pCRC, and other cellular elements. This intriguing terrain warrants more profound exploration in our forthcoming research endeavors.

## CONCLUSION

In this investigation, we elucidated potential molecular mechanisms contributing to LM in CRC by conducting a comprehensive single-cell multi-omics analysis. This analysis involved enrichment studies, cell communication assessments, cell trajectory evaluations, and transcription factor regulation scrutiny. Furthermore, we established a network that clarifies the role of senescence or exhausted immune cells in regulating EMT. Through the integration of machine learning algorithms, we formulated a prognostic risk model founded on markers related to senescence. Our holistic approach has brought to light the significant involvement of senescent and exhausted immune cells in LM. Moreover, our research has unveiled intriguing phenomena within the immune microenvironments of pCRC and LM, providing a strong basis for further in-depth exploration.

## Supplementary Materials

Supplementary Figures

Supplementary Tables

## References

[1] Siegel RL, Miller KD, Wagle NS, Jemal A. Cancer statistics, 2023. CA Cancer J Clin. 2023; 73:17–48. 10.3322/caac.21763 36633525

[2] Stewart CL, Warner S, Ito K, Raoof M, Wu GX, Kessler J, Kim JY, Fong Y. Cytoreduction for colorectal metastases: liver, lung, peritoneum, lymph nodes, bone, brain. When does it palliate, prolong survival, and potentially cure? Curr Probl Surg. 2018; 55:330–79. 10.1067/j.cpsurg.2018.08.004 30526930 PMC6422355

[3] Morris VK, Kennedy EB, Baxter NN, Benson AB 3rd, Cercek A, Cho M, Ciombor KK, Cremolini C, Davis A, Deming DA, Fakih MG, Gholami S, Hong TS, et al. Treatment of Metastatic Colorectal Cancer: ASCO Guideline. J Clin Oncol. 2023; 41:678–700. 10.1200/JCO.22.01690 36252154 PMC10506310

[4] Galon J, Bruni D. Approaches to treat immune hot, altered and cold tumours with combination immunotherapies. Nat Rev Drug Discov. 2019; 18:197–218. 10.1038/s41573-018-0007-y 30610226

[5] Llosa NJ, Cruise M, Tam A, Wicks EC, Hechenbleikner EM, Taube JM, Blosser RL, Fan H, Wang H, Luber BS, Zhang M, Papadopoulos N, Kinzler KW, et al. The vigorous immune microenvironment of microsatellite instable colon cancer is balanced by multiple counter-inhibitory checkpoints. Cancer Discov. 2015; 5:43–51. 10.1158/2159-8290.CD-14-0863 25358689 PMC4293246

[6] Zhao Y, Shao Q, Peng G. Exhaustion and senescence: two crucial dysfunctional states of T cells in the tumor microenvironment. Cell Mol Immunol. 2020; 17:27–35. 10.1038/s41423-019-0344-8 31853000 PMC6952436

[7] Kaiser M, Semeraro MD, Herrmann M, Absenger G, Gerger A, Renner W. Immune Aging and Immunotherapy in Cancer. Int J Mol Sci. 2021; 22:7016. 10.3390/ijms22137016 34209842 PMC8269421

[8] Wang B, Kohli J, Demaria M. Senescent Cells in Cancer Therapy: Friends or Foes? Trends Cancer. 2020; 6:838–57. 10.1016/j.trecan.2020.05.004 32482536

[9] Canino C, Mori F, Cambria A, Diamantini A, Germoni S, Alessandrini G, Borsellino G, Galati R, Battistini L, Blandino R, Facciolo F, Citro G, Strano S, et al. SASP mediates chemoresistance and tumor-initiating-activity of mesothelioma cells. Oncogene. 2012; 31:3148–63. 10.1038/onc.2011.485 22020330

[10] Laberge RM, Awad P, Campisi J, Desprez PY. Epithelial-mesenchymal transition induced by senescent fibroblasts. Cancer Microenviron. 2012; 5:39–44. 10.1007/s12307-011-0069-4 21706180 PMC3343197

[11] Milanovic M, Fan DNY, Belenki D, Däbritz JHM, Zhao Z, Yu Y, Dörr JR, Dimitrova L, Lenze D, Monteiro Barbosa IA, Mendoza-Parra MA, Kanashova T, Metzner M, et al. Senescence-associated reprogramming promotes cancer stemness. Nature. 2018; 553:96–100. 10.1038/nature25167 29258294

[12] Salminen A. Activation of immunosuppressive network in the aging process. Ageing Res Rev. 2020; 57:100998. 10.1016/j.arr.2019.100998 31838128

[13] Ruhland MK, Loza AJ, Capietto AH, Luo X, Knolhoff BL, Flanagan KC, Belt BA, Alspach E, Leahy K, Luo J, Schaffer A, Edwards JR, Longmore G, et al. Stromal senescence establishes an immunosuppressive microenvironment that drives tumorigenesis. Nat Commun. 2016; 7:11762. 10.1038/ncomms11762 27272654 PMC4899869

[14] Prieto LI, Sturmlechner I, Graves SI, Zhang C, Goplen NP, Yi ES, Sun J, Li H, Baker DJ. Senescent alveolar macrophages promote early-stage lung tumorigenesis. Cancer Cell. 2023; 41:1261–75.e6. 10.1016/j.ccell.2023.05.006 37267954 PMC10524974

[15] Angelini PD, Zacarias Fluck MF, Pedersen K, Parra-Palau JL, Guiu M, Bernadó Morales C, Vicario R, Luque-García A, Navalpotro NP, Giralt J, Canals F, Gomis RR, Tabernero J, et al. Constitutive HER2 signaling promotes breast cancer metastasis through cellular senescence. Cancer Res. 2013; 73:450–8. 10.1158/0008-5472.CAN-12-2301 23288917

[16] Demaria M, O'Leary MN, Chang J, Shao L, Liu S, Alimirah F, Koenig K, Le C, Mitin N, Deal AM, Alston S, Academia EC, Kilmarx S, et al. Cellular Senescence Promotes Adverse Effects of Chemotherapy and Cancer Relapse. Cancer Discov. 2017; 7:165–76. 10.1158/2159-8290.CD-16-0241 27979832 PMC5296251

[17] Thommen DS, Schumacher TN. T Cell Dysfunction in Cancer. Cancer Cell. 2018; 33:547–62. 10.1016/j.ccell.2018.03.012 29634943 PMC7116508

[18] Crespo J, Sun H, Welling TH, Tian Z, Zou W. T cell anergy, exhaustion, senescence, and stemness in the tumor microenvironment. Curr Opin Immunol. 2013; 25:214–21. 10.1016/j.coi.2012.12.003 23298609 PMC3636159

[19] Jiang Y, Li Y, Zhu B. T-cell exhaustion in the tumor microenvironment. Cell Death Dis. 2015; 6:e1792. 10.1038/cddis.2015.162 26086965 PMC4669840

[20] Lin D, Zhai X, Qi X, Zhou Q, Liu Y, Lin Y, Liu J. Senescent cancer-associated fibroblasts facilitate tumor associated neutrophil recruitment suppressing tumor immunity. J Transl Med. 2024; 22:231. 10.1186/s12967-024-05017-w 38433192 PMC10909258

[21] Baessler A, Vignali DAA. T Cell Exhaustion. Annu Rev Immunol. 2024. [Epub ahead of print]. 10.1146/annurev-immunol-090222-110914 38166256

[22] Che LH, Liu JW, Huo JP, Luo R, Xu RM, He C, Li YQ, Zhou AJ, Huang P, Chen YY, Ni W, Zhou YX, Liu YY, et al. A single-cell atlas of liver metastases of colorectal cancer reveals reprogramming of the tumor microenvironment in response to preoperative chemotherapy. Cell Discov. 2021; 7:80. 10.1038/s41421-021-00312-y 34489408 PMC8421363

[23] Li J, Wu C, Hu H, Qin G, Wu X, Bai F, Zhang J, Cai Y, Huang Y, Wang C, Yang J, Luan Y, Jiang Z, et al. Remodeling of the immune and stromal cell compartment by PD-1 blockade in mismatch repair-deficient colorectal cancer. Cancer Cell. 2023; 41:1152–69.e7. 10.1016/j.ccell.2023.04.011 37172580

[24] Satija R, Farrell JA, Gennert D, Schier AF, Regev A. Spatial reconstruction of single-cell gene expression data. Nat Biotechnol. 2015; 33:495–502. 10.1038/nbt.3192 25867923 PMC4430369

[25] Korsunsky I, Millard N, Fan J, Slowikowski K, Zhang F, Wei K, Baglaenko Y, Brenner M, Loh PR, Raychaudhuri S. Fast, sensitive and accurate integration of single-cell data with Harmony. Nat Methods. 2019; 16:1289–96. 10.1038/s41592-019-0619-0 31740819 PMC6884693

[26] Zappia L, Oshlack A. Clustering trees: a visualization for evaluating clusterings at multiple resolutions. Gigascience. 2018; 7:giy083. 10.1093/gigascience/giy083 30010766 PMC6057528

[27] Becht E, McInnes L, Healy J, Dutertre CA, Kwok IWH, Ng LG, Ginhoux F, Newell EW. Dimensionality reduction for visualizing single-cell data using UMAP. Nat Biotechnol. 2018. [Epub ahead of print]. 10.1038/nbt.4314 30531897

[28] Aran D, Looney AP, Liu L, Wu E, Fong V, Hsu A, Chak S, Naikawadi RP, Wolters PJ, Abate AR, Butte AJ, Bhattacharya M. Reference-based analysis of lung single-cell sequencing reveals a transitional profibrotic macrophage. Nat Immunol. 2019; 20:163–72. 10.1038/s41590-018-0276-y 30643263 PMC6340744

[29] Qiu X, Mao Q, Tang Y, Wang L, Chawla R, Pliner HA, Trapnell C. Reversed graph embedding resolves complex single-cell trajectories. Nat Methods. 2017; 14:979–82. 10.1038/nmeth.4402 28825705 PMC5764547

[30] Cao J, Spielmann M, Qiu X, Huang X, Ibrahim DM, Hill AJ, Zhang F, Mundlos S, Christiansen L, Steemers FJ, Trapnell C, Shendure J. The single-cell transcriptional landscape of mammalian organogenesis. Nature. 2019; 566:496–502. 10.1038/s41586-019-0969-x 30787437 PMC6434952

[31] Trapnell C, Cacchiarelli D, Grimsby J, Pokharel P, Li S, Morse M, Lennon NJ, Livak KJ, Mikkelsen TS, Rinn JL. The dynamics and regulators of cell fate decisions are revealed by pseudotemporal ordering of single cells. Nat Biotechnol. 2014; 32:381–6. 10.1038/nbt.2859 24658644 PMC4122333

[32] Korotkevich G, Sukhov V, Budin N, Shpak B, Artyomov MN, Sergushichev A. Fast gene set enrichment analysis. bioRxiv. 2021; 060012. 10.1101/060012

[33] Wu Y, Yang S, Ma J, Chen Z, Song G, Rao D, Cheng Y, Huang S, Liu Y, Jiang S, Liu J, Huang X, Wang X, et al. Spatiotemporal Immune Landscape of Colorectal Cancer Liver Metastasis at Single-Cell Level. Cancer Discov. 2022; 12:134–53. 10.1158/2159-8290.CD-21-0316 34417225

[34] Jin S, Guerrero-Juarez CF, Zhang L, Chang I, Ramos R, Kuan CH, Myung P, Plikus MV, Nie Q. Inference and analysis of cell-cell communication using CellChat. Nat Commun. 2021; 12:1088. 10.1038/s41467-021-21246-9 33597522 PMC7889871

[35] Aibar S, González-Blas CB, Moerman T, Huynh-Thu VA, Imrichova H, Hulselmans G, Rambow F, Marine JC, Geurts P, Aerts J, van den Oord J, Atak ZK, Wouters J, Aerts S. SCENIC: single-cell regulatory network inference and clustering. Nat Methods. 2017; 14:1083–6. 10.1038/nmeth.4463 28991892 PMC5937676

[36] Vasaikar SV, Deshmukh AP, den Hollander P, Addanki S, Kuburich NA, Kudaravalli S, Joseph R, Chang JT, Soundararajan R, Mani SA. EMTome: a resource for pan-cancer analysis of epithelial-mesenchymal transition genes and signatures. Br J Cancer. 2021; 124:259–69. 10.1038/s41416-020-01178-9 33299129 PMC7782839

[37] Zhang B, Horvath S. A general framework for weighted gene co-expression network analysis. Stat Appl Genet Mol Biol. 2005; 4:Article17. 10.2202/1544-6115.1128 16646834

[38] Langfelder P, Horvath S. WGCNA: an R package for weighted correlation network analysis. BMC Bioinformatics. 2008; 9:559. 10.1186/1471-2105-9-559 19114008 PMC2631488

[39] Smith JJ, Deane NG, Wu F, Merchant NB, Zhang B, Jiang A, Lu P, Johnson JC, Schmidt C, Bailey CE, Eschrich S, Kis C, Levy S, et al. Experimentally derived metastasis gene expression profile predicts recurrence and death in patients with colon cancer. Gastroenterology. 2010; 138:958–68. 10.1053/j.gastro.2009.11.005 19914252 PMC3388775

[40] Chen DT, Hernandez JM, Shibata D, McCarthy SM, Humphries LA, Clark W, Elahi A, Gruidl M, Coppola D, Yeatman T. Complementary strand microRNAs mediate acquisition of metastatic potential in colonic adenocarcinoma. J Gastrointest Surg. 2012; 16:905–12. 10.1007/s11605-011-1815-0 22362069 PMC6753785

[41] Tripathi MK, Deane NG, Zhu J, An H, Mima S, Wang X, Padmanabhan S, Shi Z, Prodduturi N, Ciombor KK, Chen X, Washington MK, Zhang B, Beauchamp RD. Nuclear factor of activated T-cell activity is associated with metastatic capacity in colon cancer. Cancer Res. 2014; 74:6947–57. 10.1158/0008-5472.CAN-14-1592 25320007 PMC4252979

[42] Marisa L, de Reyniès A, Duval A, Selves J, Gaub MP, Vescovo L, Etienne-Grimaldi MC, Schiappa R, Guenot D, Ayadi M, Kirzin S, Chazal M, Fléjou JF, et al. Gene expression classification of colon cancer into molecular subtypes: characterization, validation, and prognostic value. PLoS Med. 2013; 10:e1001453. 10.1371/journal.pmed.1001453 23700391 PMC3660251

[43] Liu X, Li X, Wei H, Liu Y, Li N. Mast cells in colorectal cancer tumour progression, angiogenesis, and lymphangiogenesis. Front Immunol. 2023; 14:1209056. 10.3389/fimmu.2023.1209056 37497234 PMC10366593

[44] Wang C, Long Q, Fu Q, Xu Q, Fu D, Li Y, Gao L, Guo J, Zhang X, Lam EW, Campisi J, Sun Y. Targeting epiregulin in the treatment-damaged tumor microenvironment restrains therapeutic resistance. Oncogene. 2022; 41:4941–59. 10.1038/s41388-022-02476-7 36202915 PMC9630100

[45] Tabula Muris Consortium. A single-cell transcriptomic atlas characterizes ageing tissues in the mouse. Nature. 2020; 583:590–5. 10.1038/s41586-020-2496-1 32669714 PMC8240505

[46] Xiao FH, Chen XQ, Yu Q, Ye Y, Liu YW, Yan D, Yang LQ, Chen G, Lin R, Yang L, Liao X, Zhang W, Zhang W, et al. Transcriptome evidence reveals enhanced autophagy-lysosomal function in centenarians. Genome Res. 2018; 28:1601–10. 10.1101/gr.220780.117 30352807 PMC6211641

[47] Welch C, Santra MK, El-Assaad W, Zhu X, Huber WE, Keys RA, Teodoro JG, Green MR. Identification of a protein, G0S2, that lacks Bcl-2 homology domains and interacts with and antagonizes Bcl-2. Cancer Res. 2009; 69:6782–9. 10.1158/0008-5472.CAN-09-0128 19706769 PMC2841785

[48] Kang C, Elledge SJ. How autophagy both activates and inhibits cellular senescence. Autophagy. 2016; 12:898–9. 10.1080/15548627.2015.1121361 27129029 PMC4854549

[49] Tower J. Programmed cell death in aging. Ageing Res Rev. 2015; 23:90–100. 10.1016/j.arr.2015.04.002 25862945 PMC4480161

[50] Economopoulou MA, Fragoulis EG, Sideris DC. Molecular cloning and characterization of the human RNase kappa, an ortholog of Cc RNase. Nucleic Acids Res. 2007; 35:6389–98. 10.1093/nar/gkm718 17881363 PMC2095791

[51] Bancaro N, Calì B, Troiani M, Elia AR, Arzola RA, Attanasio G, Lai P, Crespo M, Gurel B, Pereira R, Guo C, Mosole S, Brina D, et al. Apolipoprotein E induces pathogenic senescent-like myeloid cells in prostate cancer. Cancer Cell. 2023; 41:602–19.e11. 10.1016/j.ccell.2023.02.004 36868226

[52] Dolina JS, Van Braeckel-Budimir N, Thomas GD, Salek-Ardakani S. CD8^+^ T Cell Exhaustion in Cancer. Front Immunol. 2021; 12:715234. 10.3389/fimmu.2021.715234 34354714 PMC8330547

[53] Grootaert C, Van de Wiele T, Verstraete W, Bracke M, Vanhoecke B. Angiopoietin-like protein 4: health effects, modulating agents and structure-function relationships. Expert Rev Proteomics. 2012; 9:181–99. 10.1586/epr.12.12 22462789

[54] Hübers C, Abdul Pari AA, Grieshober D, Petkov M, Schmidt A, Messmer T, Heyer CM, Schölch S, Kapel SS, Gengenbacher N, Singhal M, Schieb B, Fricke C, et al. Primary tumor-derived systemic nANGPTL4 inhibits metastasis. J Exp Med. 2023; 220:e20202595. 10.1084/jem.20202595 36269299 PMC9595206

[55] Wang Q, Oliver-Williams C, Raitakari OT, Viikari J, Lehtimäki T, Kähönen M, Järvelin MR, Salomaa V, Perola M, Danesh J, Kettunen J, Butterworth AS, Holmes MV, Ala-Korpela M. Metabolic profiling of angiopoietin-like protein 3 and 4 inhibition: a drug-target Mendelian randomization analysis. Eur Heart J. 2021; 42:1160–9. 10.1093/eurheartj/ehaa972 33351885 PMC7982288

[56] Mizuno S, Seishima R, Yamasaki J, Hattori K, Ogiri M, Matsui S, Shigeta K, Okabayashi K, Nagano O, Li L, Kitagawa Y. Angiopoietin-like 4 promotes glucose metabolism by regulating glucose transporter expression in colorectal cancer. J Cancer Res Clin Oncol. 2022; 148:1351–61. 10.1007/s00432-022-03960-z 35195748 PMC11800850

[57] Wei C, Yang C, Wang S, Shi D, Zhang C, Lin X, Liu Q, Dou R, Xiong B. Crosstalk between cancer cells and tumor associated macrophages is required for mesenchymal circulating tumor cell-mediated colorectal cancer metastasis. Mol Cancer. 2019; 18:64. 10.1186/s12943-019-0976-4 30927925 PMC6441214

[58] Funamizu N, Hu C, Lacy C, Schetter A, Zhang G, He P, Gaedcke J, Ghadimi MB, Ried T, Yfantis HG, Lee DH, Subleski J, Chan T, et al. Macrophage migration inhibitory factor induces epithelial to mesenchymal transition, enhances tumor aggressiveness and predicts clinical outcome in resected pancreatic ductal adenocarcinoma. Int J Cancer. 2013; 132:785–94. 10.1002/ijc.27736 22821831 PMC3488363

[59] Park GB, Chung YH, Gong JH, Jin DH, Kim D. GSK-3β-mediated fatty acid synthesis enhances epithelial to mesenchymal transition of TLR4-activated colorectal cancer cells through regulation of TAp63. Int J Oncol. 2016; 49:2163–72. 10.3892/ijo.2016.3679 27599658

[60] Wang Y, Shi J, Chai K, Ying X, Zhou BP. The Role of Snail in EMT and Tumorigenesis. Curr Cancer Drug Targets. 2013; 13:963–72. 10.2174/15680096113136660102 24168186 PMC4004763

[61] Dilshat R, Fock V, Kenny C, Gerritsen I, Lasseur RMJ, Travnickova J, Eichhoff OM, Cerny P, Möller K, Sigurbjörnsdóttir S, Kirty K, Einarsdottir BÓ, Cheng PF, et al. MITF reprograms the extracellular matrix and focal adhesion in melanoma. Elife. 2021; 10:e63093. 10.7554/eLife.63093 33438577 PMC7857731

[62] Pompili S, Vetuschi A, Latella G, Smakaj A, Sferra R, Cappariello A. PPAR-Gamma Orchestrates EMT, AGE, and Cellular Senescence Pathways in Colonic Epithelium and Restrains the Progression of IBDs. Int J Mol Sci. 2023; 24:8952. 10.3390/ijms24108952 37240299 PMC10219383

[63] Yang S, Xu L, Yang T, Wang F. High-mobility group box-1 and its role in angiogenesis. J Leukoc Biol. 2014; 95:563–74. 10.1189/jlb.0713412 24453275

[64] Mishra VK, Subramaniam M, Kari V, Pitel KS, Baumgart SJ, Naylor RM, Nagarajan S, Wegwitz F, Ellenrieder V, Hawse JR, Johnsen SA. Krüppel-like Transcription Factor KLF10 Suppresses TGFβ-Induced Epithelial-to-Mesenchymal Transition via a Negative Feedback Mechanism. Cancer Res. 2017; 77:2387–400. 10.1158/0008-5472.CAN-16-2589 28249899 PMC5445903

