# Supplementary material for: Immune cell senescence and exhaustion promote the occurrence of liver metastasis in colorectal cancer by regulating epithelial-mesenchymal transition
Source: Aging (Albany NY). 2024 Apr 26;16(9):7704–32. doi: 10.18632/aging.205778 (PMC11132022; doi:10.18632/aging.205778)
Supplement: Supplementary Figures [file aging-16-205778-s001.pdf]

SUPPLEMENTARY FIGURES

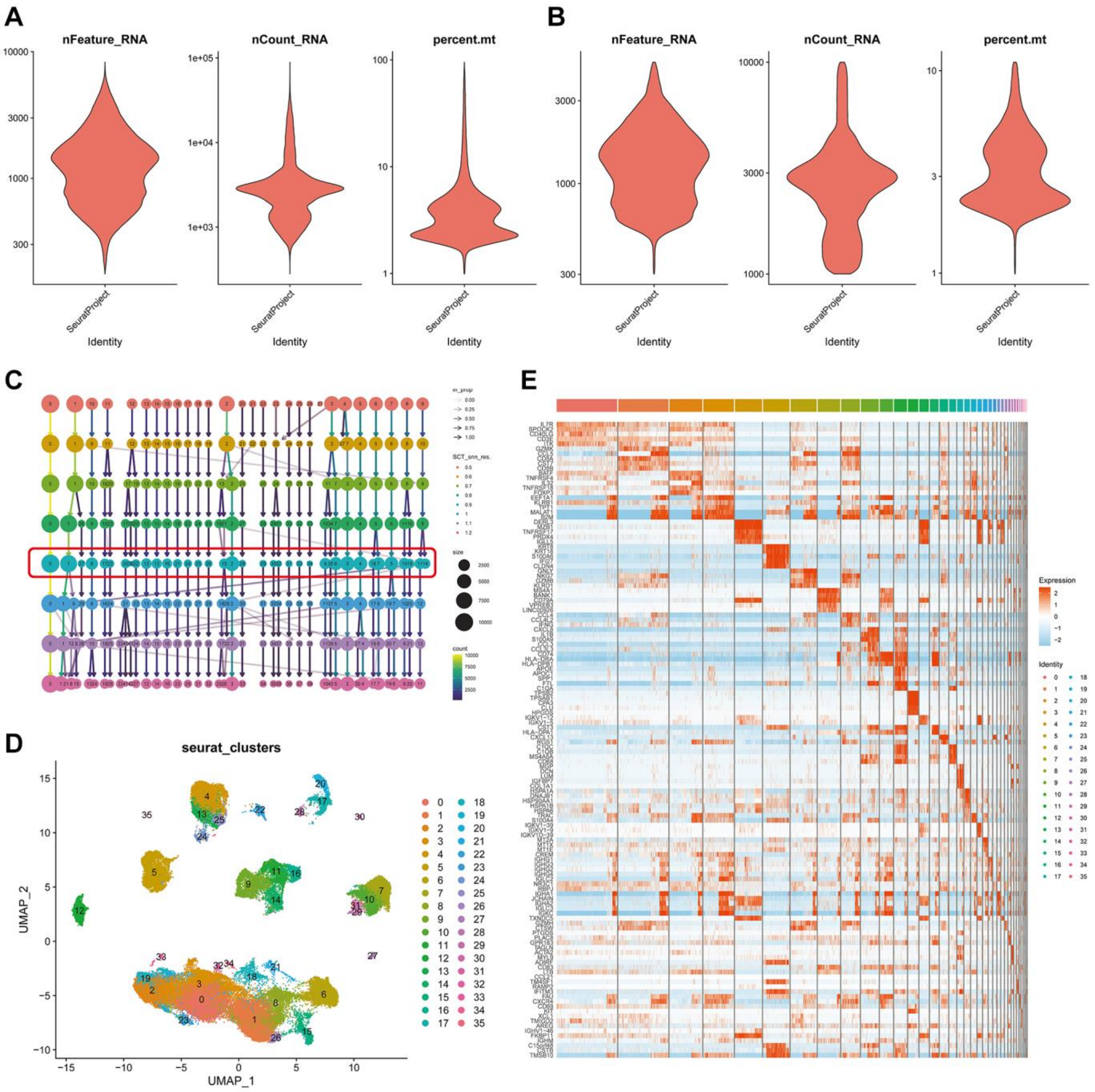

Supplementary Figure 1. Quality control and dimensionality reduction clustering of primary colorectal cancer scRNA-Seq data. (**A**, **B**) Data features before (**A**) and after (**B**) quality control of scRNA-Seq data. (**C**) Clustree dendrogram demonstrating dimensionality reduction clustering at varying resolutions (0.5–1.2). (**D**) Dimensionality reduction clustering of all cells utilizing the UMAP plot at the appropriate resolution. (**E**) Heatmap presenting the gene expression levels of the top 5 differentially expressed genes across distinct clusters.

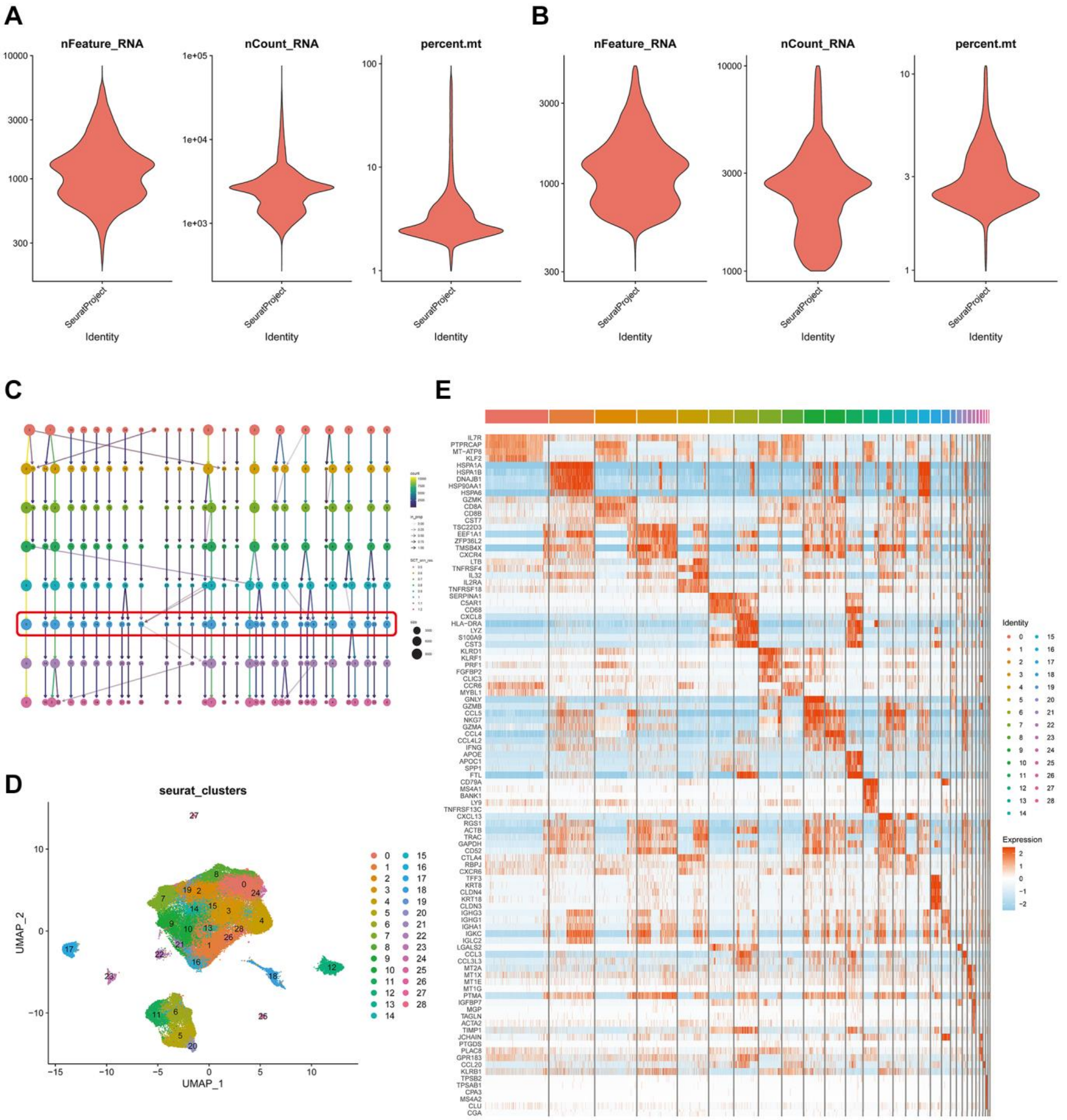

Supplementary Figure 2. Quality control and dimensionality reduction clustering of liver metastasis scRNA-Seq data. (**A**, **B**) Data features before (**A**) and after (**B**) quality control of scRNA-Seq data. (**C**) Clustree dendrogram showcasing dimensionality reduction clustering at different resolutions (0.5–1.2). (**D**) Dimensionality reduction clustering of all cells utilizing the UMAP plot at the optimal resolution. (**E**) Heatmap illustrating the gene expression levels of the top 5 differentially expressed genes within different clusters.

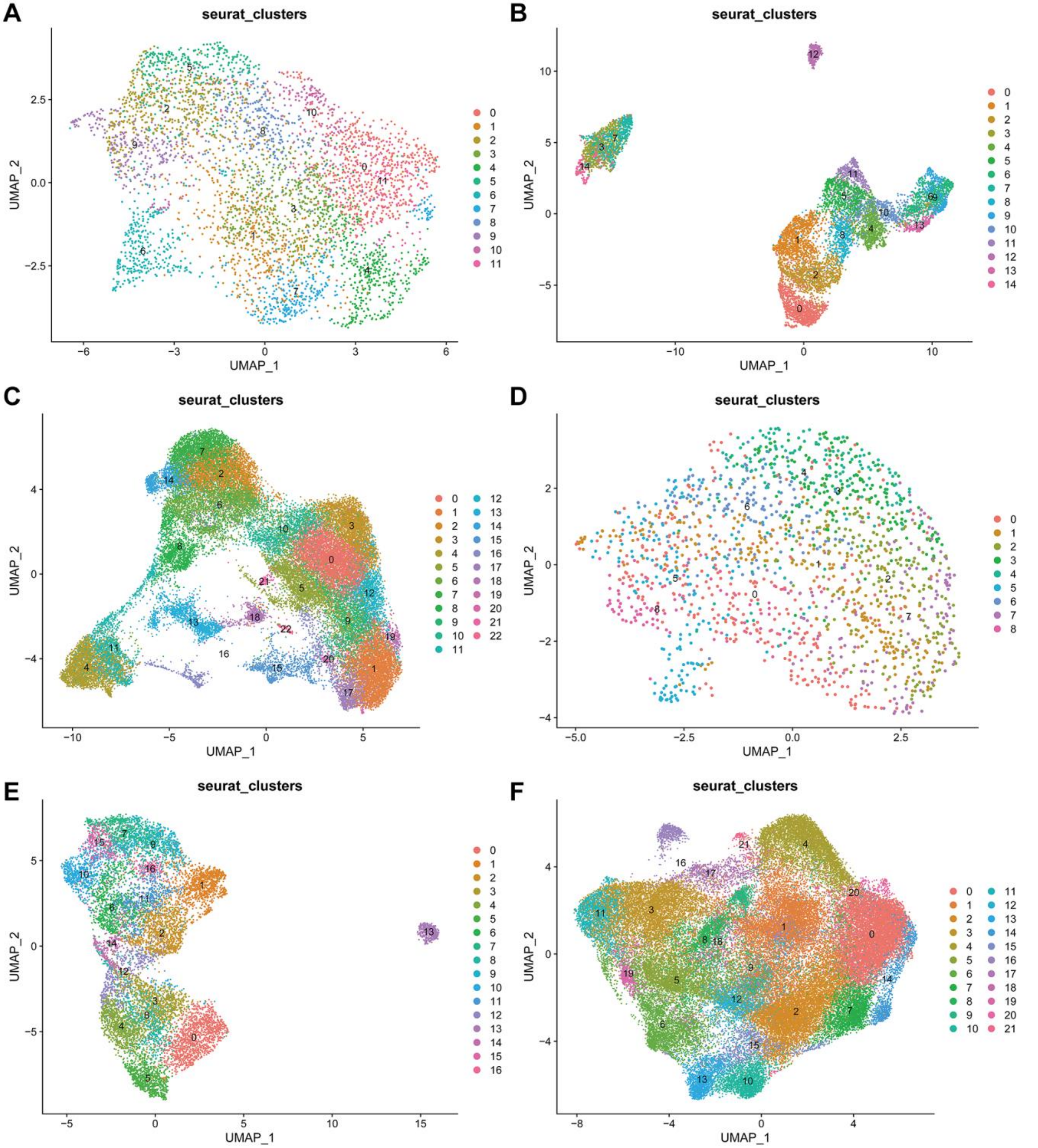

Supplementary Figure 3. Dimensionality reduction clustering results of main cell types. (**A**–**C**) UMAP plots for dimensionality reduction clustering of epithelial cells (**A**), myeloid cells (**B**), and NK/T cells (**C**) in primary colorectal cancer tissues. (**D**–**F**) UMAP plots for dimensionality reduction clustering of epithelial cells (**D**), myeloid cells (**E**), and NK/T cells (**F**) in liver metastasis tissues.

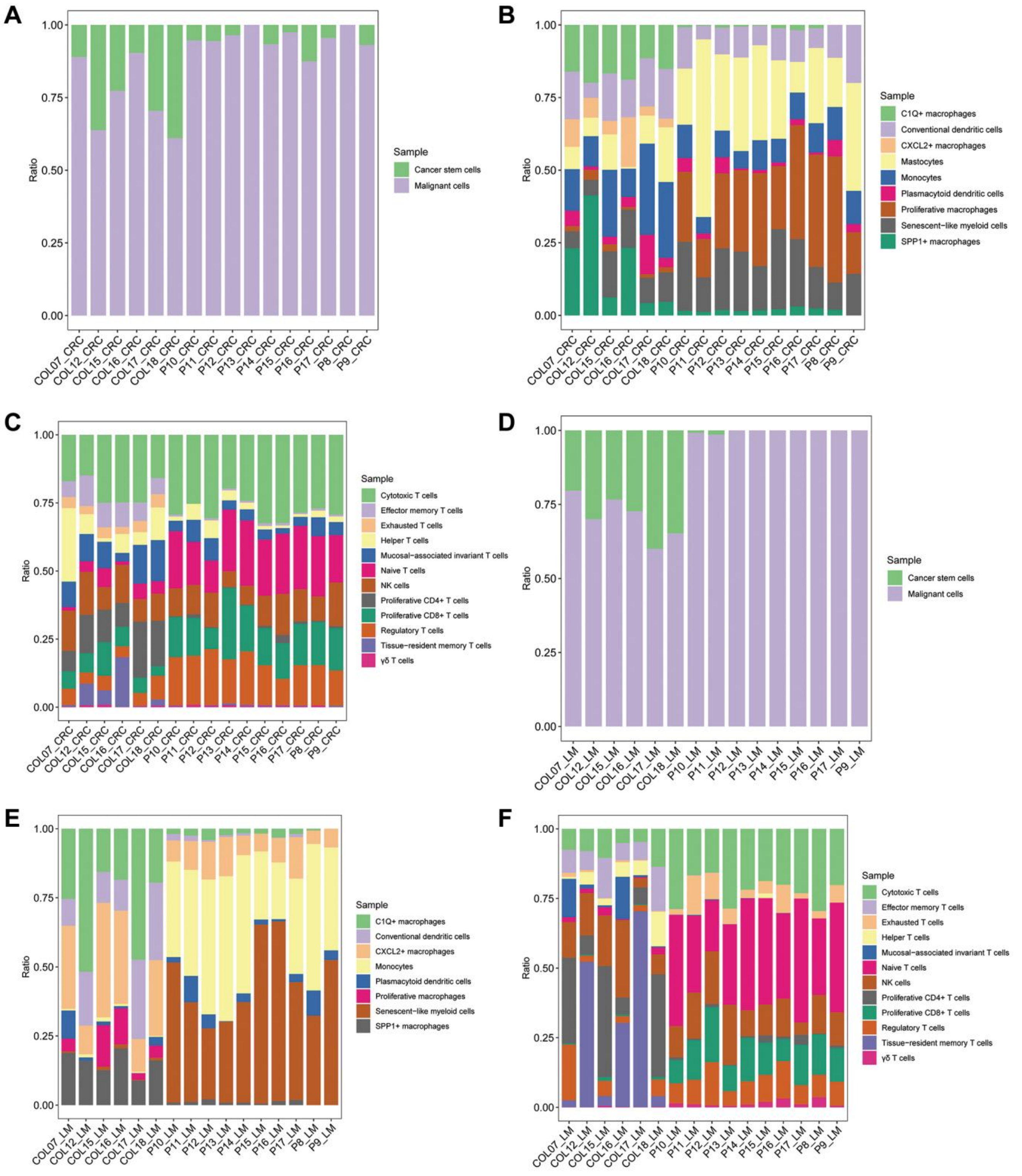

Supplementary Figure 4. Proportion plots of main cell types and their subtypes. (**A**–**C**) Proportion plots representing the distribution of cell subtypes within epithelial cells (**A**), myeloid cells (**B**), and NK/T cells (**C**) in primary colorectal cancer tissues. (**D**–**F**) Proportion plots illustrating the distribution of cell subtypes within epithelial cells (**D**), myeloid cells (**E**), and NK/T cells (**F**) in liver metastasis tissues.
