# Supplementary material for: Immune cell senescence and exhaustion promote the occurrence of liver metastasis in colorectal cancer by regulating epithelial-mesenchymal transition
Source: Aging (Albany NY). 2024 Apr 26;16(9):7704–32. doi: 10.18632/aging.205778 (PMC11132022; doi:10.18632/aging.205778)
Supplement: Supplementary Tables [file aging-16-205778-s002.pdf]

SUPPLEMENTARY TABLES

Supplementary Table 1. siRNA sequences and primer sequences.

	Sequencing
si-SDC1	Sense: CCUGCCGCAAAUUGUGGCUACUAAU<br>Antisense: AUUAGUAGCCACAAUUUGCGGCAGG
si-SDC4	Sense: GACCUCCUAGAAGGCCGAUACUUCU<br>Antisense: AGAAGUAUCGGCCUUCUAGGAGGUC
CDH1	Forward: 5′-CGAGAGCTACACGTTCACGG-3′<br>Reverse: 5′-GGGTGTCGAGGGAAAAATAGG-3′
CDH2	Forward: 5′-AGCCAACCTTAACTGAGGAGT-3′<br>Reverse: 5′-GGCAAGTTGATTGGAGGGATG-3′
VIM	Forward: 5′-GACGCCATCAACACCGAGTT-3′<br>Reverse: 5′-CTTTGTCGTTGGTTAGCTGGT-3′
β-Actin	Forward: 5′-AGGATGCAGAAGGAGATCAC-3′<br>Reverse: 5′-TGTAACGCAACTAAGTCATAG-3′

Supplementary Table 2. Reverse transcription reaction mixture.

Reagents	Quantities/μL
5 × gDNA Eraser Buffer	2
gDNA Eraser	1
Total RNA	4
RNase Free dH2O	3
Total	10

Supplementary Table 3. Reverse transcription reaction system.

Reagents	Quantities/μL
Reverse transcription reaction mixture	10
PrimeScript RT Enzyme Mix I	1
RT Primer Mix	4
5×PrimeScript Buffer 2	4
RNase Free dH2O	1
Total	20

Supplementary Table 4. qRT-PCR reaction system.

Reagents	Quantities/μL
SYBR Premix Ex Taq™II	5
Forward Primer (10 μM)	0.2
Reference Primer (10 μM)	0.2
Template DNA	1
ddH_2O	3.6
Total	10
